# Prevalence of Human Papillomavirus and Genotype Distribution in Chinese Men: A Systematic Review and Meta‐Analysis

**DOI:** 10.1002/cam4.70686

**Published:** 2025-02-17

**Authors:** Yifan Li, Fan Zhao, Dan Wu, Chuanyu Qin, Yajiao Lu, Ying Yang, Hairong Wang, Chunlei Lu, Shengyue Qiu, Wenwen Jiang, Yuxiu Yan, Xianyi Geng, Hongding Rong, Na Ji, Ning Lv, Yue Li, Jing Li

**Affiliations:** ^1^ West China School of Public Health and West China Fourth Hospital Sichuan University Chengdu China; ^2^ Department of Social Medicine and Health Education School of Public Health, Nanjing Medical University Nanjing China

**Keywords:** China, distribution, human papillomavirus, male, systematic review

## Abstract

**Background:**

Human papillomavirus (HPV) vaccination represents a cost‐effective strategy for preventing HPV‐related diseases across genders. However, the HPV vaccine has not been approved for mainland Chinese males, and the comprehensive epidemiological landscape of HPV among Chinese males from mainland China is limited.

**Methods:**

This study aimed to address this gap by examining HPV infection data in Chinese males from January 2012 to September 2024. Four English databases (Web of Science, Embase, Medline, and Cochrane Library) and four Chinese databases (CNKI, VIP, Wanfang, and SinoMed) were systematically reviewed. Random effect models assessed pooled HPV prevalence, and subgroup analyses were conducted based on population (outpatients vs. health checkups). Genotype‐specific HPV positivity was calculated.

**Results:**

A total of 296 studies were included, encompassing 199,233 outpatients and 16,452 health checkups. HPV prevalence was 52.45% among outpatients, with the most prevalent subtypes being HPV 6 (19.06%), 11 (13.71%), and 16 (8.29%). Among health checkups, HPV prevalence was 7.89%, with the highest prevalence subtypes being HPV 16 (3.66%), 52 (1.37%), and 58 (1.19%). Among male patients diagnosed with cancer, HPV 16 (18.50%) and 18 (5.33%) were the most common subtypes, and HPV prevalence was 82.11% among the HIV‐positive MSM population.

**Conclusion:**

The high prevalence of HPV among Chinese males, particularly among outpatients and the HIV‐positive MSM population, underscores the urgent need for targeted prevention strategies. The common subtypes identified in this analysis highlight the potential benefits of introducing HPV prophylactic vaccines to Chinese males, which could significantly reduce the burden of HPV‐related diseases across the population.

## 1 Introduction

Human papillomavirus (HPV) infection is the most prevalent sexually transmitted infection, significantly contributing to various conditions in both genders [1, 2]. Despite a wealth of research on HPV prevalence among women, studies focusing on men are notably limited—largely due to the predominant concern regarding cervical cancer. However, HPV infections carry substantial risks for males as well. In 2020, approximately 561,295 cases of HPV‐related cancers were diagnosed globally in men [3], with about 10% of these cases occurring in Chinese males [4]. Notably, laryngeal, penile, and anal cancers represented 16.1%, 12.8%, and 11.5% of these diagnoses, respectively [4]. This alarming burden underscores the urgent need for targeted prevention strategies for men.

HPV vaccination has proven to be the most cost‐effective strategy for preventing HPV‐related diseases across genders [5, 6, 7]. Although over 100 countries have integrated the HPV vaccine into national immunization programs for girls [8], the inclusion of males remains limited, particularly in mainland China, where gender‐neutral vaccination is not yet widely adopted [8]. Recent discourse advocating for gender‐neutral HPV vaccination highlights a global shift toward more comprehensive vaccine coverage [9, 10], with increasing interest in developing HPV vaccines specifically for men.

Research on HPV prevalence and genotype distribution in men remains limited, often hindered by small sample sizes, inconsistent methodologies, and an overwhelming focus on hospital‐based studies [11]. As a result, no consensus has been reached regarding which genotypes are more likely to cause diseases in males and which genotypes are more preventable through vaccination. Given men's critical role in HPV transmission [12], analyzing data from the male population is essential for informing effective prevention strategies for all genders. This meta‐analysis considered mainly two study populations for men: data from outpatients and health checkups. Understanding the prevalence and distribution of HPV types among outpatients is vital, as this group is more likely to present with HPV‐related diseases, such as genital warts and cancers. Analyzing this subgroup will provide insights into the specific HPV genotypes associated with these diseases and will help guide the development of more targeted vaccination and treatment strategies. Furthermore, studying health checkup populations, which are more representative of the general male population, provides a clearer picture of the overall HPV disease burden. This group offers a broader perspective on the distribution of HPV types and can help to compare infection patterns with those found in females, thereby providing valuable information on gender‐specific prevalence and potential vaccine effectiveness.

This study aims to systematically review and synthesize data from studies published over the past decade to elucidate the prevalence and genotype distribution of HPV among Chinese males. By comparing data from outpatients and health checkups, this meta‐analysis seeks to provide pivotal insights that will guide future vaccination efforts and support the development of evidence‐based, gender‐neutral HPV vaccination programs in China and other regions with similar demographics and healthcare challenges.

## 2 Methods

We reported this systematic review and meta‐analysis in accordance with the PRISMA guidelines [13]. This study was under the number CRD42022337643 in the International Prospective Register of Systematic Reviews (PROSPERO).

### 2.1 Search Strategy and Inclusion Criteria

Our search strategy encompassed four Chinese databases (CNKI, VIP, Wanfang, and SinoMed) and four English databases (Web of Science, Medline (Ovid), Embase, and Cochrane Library). Studies published between 1 January 2012 and 25 September 2024 were included. The combined search strategy was used (“China” OR “Chinese” OR “Hong Kong” OR “Taiwan” OR “Macau”) AND (“human papillomavirus” OR “HPV” OR “human papilloma virus” OR “papillomaviridae”) AND (“male” OR “men”) to identify studies reporting HPV prevalence among men in China. For the Chinese databases, keywords were translated into Chinese. The research was conducted without any limitations on language. Detailed search strategies for each database are provided in Appendix S1. Literature was managed using Endnote X9, where duplicates were automatically recognized and removed, with any remaining duplicates manually removed after a double‐check.

Studies that meet the following criteria were included: (1) participants were Chinese males; (2) studies reported HPV infection rates and/or HPV genotype distribution; (3) laboratory assays and sampling sites were clearly described; (4) the studies were conducted in mainland China, Hong Kong, Macau, or Taiwan; (5) studies were published between January 1, 2012 and September 25, 2024; and (6) studies were peer‐reviewed, excluding review articles and conference abstracts. Participants who were included in multiple studies were only enrolled once, and data from articles with the largest sample size and/or the most detailed information being utilized when sample sizes were the same. The literature search and inclusion assessment were carried out independently by two authors (F.Z., Y.L.). Discrepancies were resolved through consensus and, when necessary, consultation with an experienced third reviewer (J.L.).

### 2.2 Data Extraction

The primary information extracted included the identification of publication, study region, participant descriptions, study design, sample size, data collection period, the sampling site, sample type, HPV detection method, and HPV prevalence by genotypes and age groups (Appendix S1). The information was extracted concurrently by multiple authors and reviewed by Y.L. If only primary data (i.e., sample size and number of HPV infections) was provided, it was used to estimate prevalence. Any discrepancies in data extraction were resolved through consensus.

### 2.3 Quality Assessment

In this systematic review, the methodology quality was evaluated using the Agency for Healthcare Research and Quality (AHRQ) checklist for cross sectional/prevalence studies [14]. Two independent reviewers rigorously assessed the quality of studies in terms of accuracy and generalizability. For enhanced clarity and cohesion, the AHRQ scores were categorized into three quality groups: 0–3 (low quality), 4–7 (moderate quality), and 8–11 (high quality), following the categorization proposed by Cabral et al. [15]. Detailed information regarding the quality assessment is provided in Appendix S1.

### 2.4 Statistical Analysis

The number of males tested served as the denominator for reporting each prevalence. Due to inconsistent reporting across studies, denominators varied for different outcomes (e.g., any HPV‐positive pooled prevalence, any HR‐HPV positive pooled prevalence, etc.). Pooled prevalence was combined without distinguishing between HPV detection methods and sampling sites. When a single population reported multiple sampling sites, results with the highest prevalence rate were utilized. HPV positivity across age groups was estimated based on studies reporting both the number of males tested and any type of infected male within each group. Age grouping was selected to best align with available data. We performed subgroup analyses of outpatients and health checkups, stratified by different regions [16], sampling sites, sampling types, men who have sex with men (MSM) (outpatients only), cancer status (outpatients only), and sample size [17]. These variables were also included in univariate meta‐regression (Appendix S1). For studies reporting multiple population characteristics or sampling sites, results were separated to pool subgroup data.

The pooled prevalence of HPV and its 95% confidence intervals (CIs) among Chinese males was estimated using random‐effects models. The DerSimonian–Laird estimator was employed to assess variance across included studies, and the Freeman‐Tukey Double Arcsine Transformation was used to stabilize variance. Heterogeneity between studies was measured using Higgins' *I* ^2^ statistics and Cochran's *Q* test. Additionally, meta‐regression and subgroup analysis were conducted to explore the potential causes of heterogeneity. Sensitivity analysis assessed the robustness of findings by (1) leave‐one‐out method (omitting one study at a time and calculating the pooled prevalence for the remaining studies); (2) excluding studies with a high HPV prevalence population (HIV positive MSM) among outpatients. Publication bias was assessed using Egger's test and funnel plots. All analyses were two‐sided at a 0.05 significance level, conducted using Stata/SE 15.0.

## 3 Results

**FIGURE 1:**
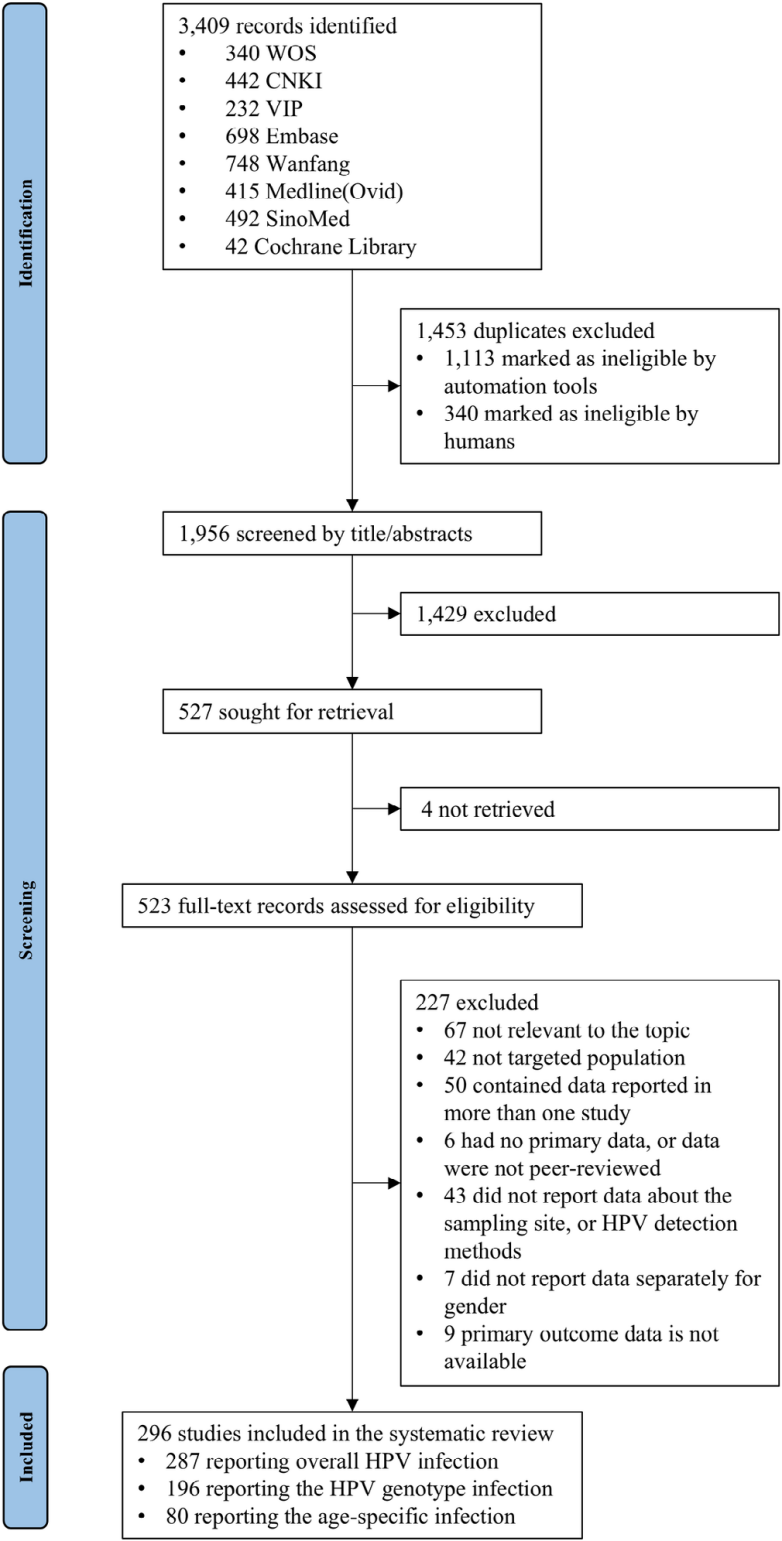
Flow chart of studies selection.

Out of 3409 identified records, 296 studies involving a total of 215,685 males met the inclusion criteria for the final systematic review (Figure 1). Among these studies, 287 (*n* = 209,730) reported HPV infections, 196 (*n* = 145,114) reported HPV genotype distributions, and 80 (*n* = 75,581) reported age‐specific HPV infections. Most studies were cross sectional (265/296), geographically conducted in Eastern China (182/296). The study population primarily consisted of hospital‐based outpatients (271/296). Specimens were predominantly collected from the anal (122/296) (Appendix S1). The detailed information on the characteristics of the included studies is provided in Appendix S1. All 296 eligible studies were of moderate or high quality based on the AHRQ checklist (Appendix S1). Egger's test and funnel plots indicated publication bias among outpatient males (Appendix S1). Evidence of heterogeneity was observed in pooled HPV prevalence and genotype distribution across studies (*I* ^2^ > 50% or *p* < 0.05). Pooled estimates remained stable when employing the leave‐one‐out method and after excluding MSM with HIV positivity (Appendix S1).

### 3.1 Overall HPV Prevalence and Genotype Distribution Among Chinese Males

As shown in Table 1, when the sampling site and specific infection genotype are not taken into consideration, the pooled HPV positivity of any HPV infection among Chinese males was 52.45% (95% CI, 49.54–55.35) in outpatients and 7.89% (95% CI, 5.18–11.06) in health checkups. For HR‐HPV, the positivity was 31.91% (95% CI, 29.40–34.48) in outpatients and 8.21% (95% CI, 3.87–13.91) in health checkups, whereas LR‐HPV positivity was 39.39% (95% CI, 35.12–43.74) and 1.85% (95% CI, 0.86–3.13), respectively. Single‐type HPV infection was more prevalent than multiple infections (30.82% vs. 21.40% in outpatients and 11.10% vs. 2.77% in health checkups) among Chinese males. In outpatients, single LR‐HPV infection was more prevalent, whereas single HR‐HPV infection was predominated in health checkups.

**TABLE 1:** HPV prevalence among Chinese males.

	Number of studies^e^	Number of males tested	Number of male HPV positive	Pooled prevalence (95% CI)	*I* ^*2*^ for heterogeneity (%)	*P* for heterogeneity
Health checkups						
Any HPV positive	25	16,452	1366	7.89 (5.18–11.06)	97.41%	< 0.001
Any HR‐HPV positive^a^	10	10,402	893	8.21 (3.87–13.91)	98.44%	< 0.001
Any LR‐HPV positive^a^	10	10,402	227	1.85 (0.86–3.13)	90.68%	< 0.001
Single‐genotype infection	6	6382	693	11.10 (6.46–16.77)	96.70%	< 0.001
Multiple genotype infection^c^	6	6382	210	2.77 (0.93–5.46)	92.81%	< 0.001
Single HR‐HPV infection^b^	4	5838	548	9.31 (3.86–16.74)	96.57%	< 0.001
Multiple HR‐HPV infection^d^	3	5408	107	0.42 (0.00–2.92)	93.50%	< 0.001
Single LR‐HPV infection^b^	4	5838	78	1.29 (0.43–2.53)	76.55%	0.01
Multiple LR‐HPV infection^d^	3	5408	1	0.00 (0.00–0.00)	0.00%	0.799
HR‐LR Mixed infection	5	5876	44	0.23 (0.00–1.10)	76.18%	0.0021
Outpatients						
Any HPV positive	273	193,278	84,350	52.45 (49.54–55.35)	99.39%	< 0.001
Any HR‐HPV positive^a^	133	84,483	25,812	31.91 (29.40–34.48)	98.36%	< 0.001
Any LR‐HPV positive^a^	111	72,167	28,179	39.39 (35.12–43.74)	99.28%	< 0.001
Single‐genotype infection	158	118,315	32,177	30.82 (29.12–32.55)	97.06%	< 0.001
Multiple genotype infection^c^	158	117,922	22,627	21.40 (19.33–23.54)	98.70%	< 0.001
Single HR‐HPV infection^b^	65	44,368	4613	10.03 (8.92–11.19)	92.84%	< 0.001
Multiple HR‐HPV infection^d^	52	32,207	1149	3.22 (2.54–3.98)	91.33%	< 0.001
Single LR‐HPV infection^b^	59	40,767	7761	19.75 (17.16–22.48)	97.69%	< 0.001
Multiple LR‐HPV infection^d^	44	28,103	1014	2.76 (1.93–3.71)	94.48%	< 0.001
HR‐LR Mixed infection	105	65,943	9659	12.97 (10.84–15.25)	98.56%	< 0.001

^a^ Any HR‐HPV/LR‐HPV positive indicates that an individual is infected with one or more HR‐HPV/LR‐HPV genotypes.

^b^ Single HR‐HPV/LR‐HPV infection implies that the individual is infected with only one HR/LR genotype.

^c^ Multiple‐genotype infection is defined as simultaneous infection of more than one HPV genotype, irrespective of these genotypes are HR or LR.

^d^ Multiple HR‐HPV/LR‐HPV infection refers to simultaneous infection with more than one HR‐HPV/LR‐HPV genotype.

^e^ The total number of studies pertaining to the two populations does not amount to 296 because of the inclusion of certain studies that encompass two distinct populations simultaneously. HR‐HPV, high‐risk human papillomavirus; LR‐HPV, low‐risk human papillomavirus.

**FIGURE 2:**
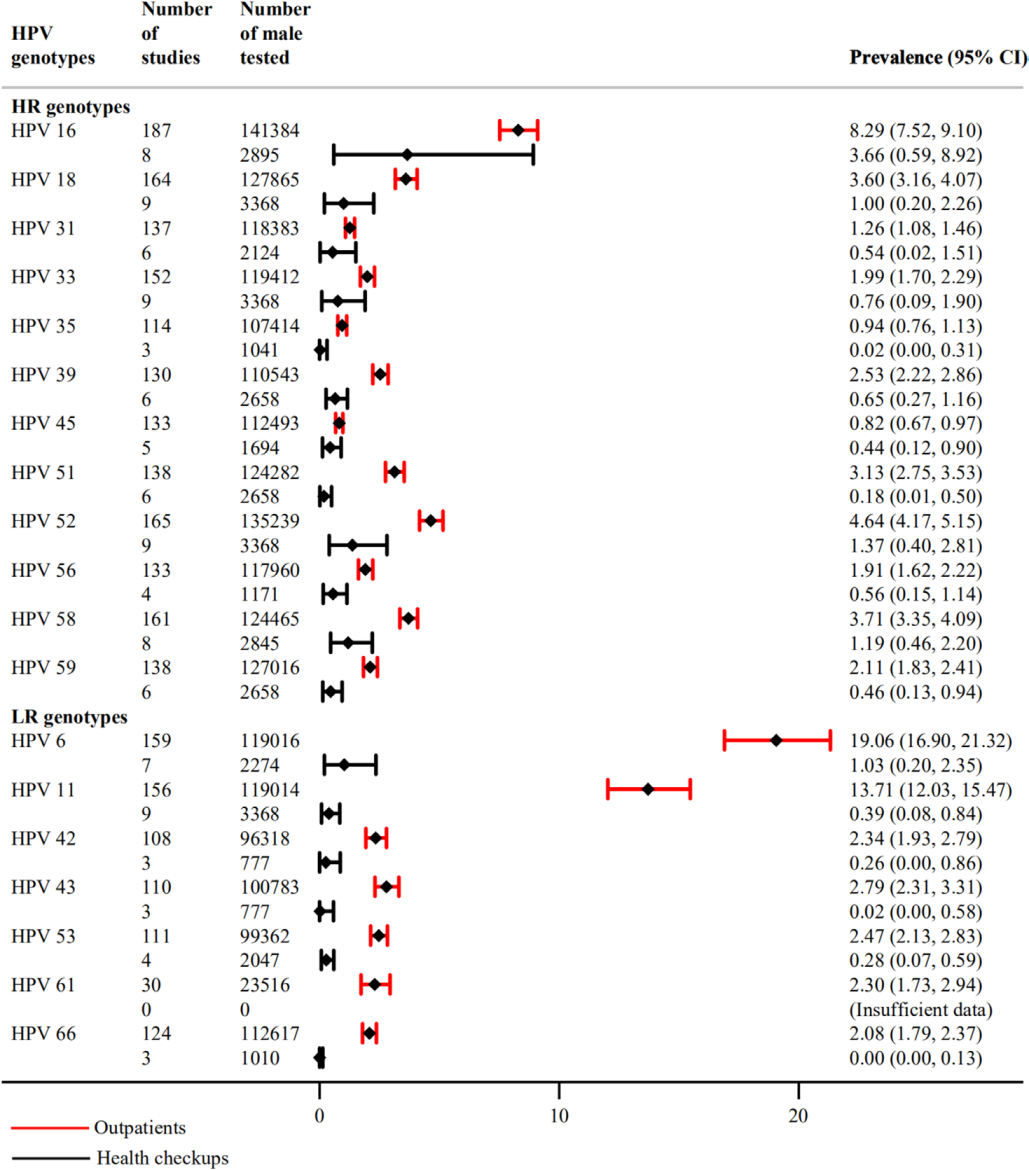
Type‐specific HPV prevalence among Chinese male outpatients and health checkups.

The forest plot in Figure 2 illustrates the top five HPV genotypes: among outpatients, these were HPV 6 (19.06%; 95% CI, 16.90–21.32), 11 (13.71%; 95% CI, 12.03–15.47), 16 (8.29%; 95% CI, 7.52–9.10), 52 (4.64%; 95% CI, 4.17–5.15), and 58 (3.71%; 95% CI, 3.35–4.09), whereas in health checkups, HPV 16 (3.66%; 95% CI, 0.59–8.92), 52 (1.37%; 95% CI, 0.40–2.81), 58 (1.19%; 95% CI, 0.46–2.20), 6 (1.03%; 95% CI, 0.20–2.35), and 18 (1.00%; 95% CI, 0.20–2.26) were the most common.

### 3.2 Stratification Variables‐Related HPV Infections Among Chinese Males

When stratified by geographical region, Central China exhibits the highest HPV prevalence among both outpatients (57.31%; 95% CI, 48.39–66.01) and health checkups (24.15%; 95% CI, 20.74–27.73) compared to other regions in China (Table 2). Notably, the genital area had the highest infection rate in health checkups (8.43%; 95% CI, 2.16–17.84) and in outpatients (66.25%; 95% CI, 60.53–71.75). Regarding sampling types, HPV prevalence is higher in tissue samples (80.98%; 95% CI, 52.30–98.31), exfoliated cells (55.25%; 95% CI, 52.20–58.29), and biopsies (42.38%; 95% CI, 30.55–54.66) among outpatients. Furthermore, studies with sample sizes under 200 reported higher pooled HPV prevalence than those with larger sample sizes (Table 2).

**TABLE 2:** Pooled HPV prevalence among Chinese males by stratified variables.

		Number of studies	Number of males tested	Number of male HPV positive	Pooled prevalence (95% CI)	*I* ^2^ for heterogeneity (%)	*P* for heterogeneity
Region							
Eastern China	Health checkups	13	6513	310	4.92 (3.31–6.81)	85.35	< 0.001
	Outpatients	169	140,830	62,824	52.48 (49.07–55.88)	99.39	< 0.001
Western China	Health checkups	8	9277	906	9.17 (3.54–16.91)	98.20	< 0.001
	Outpatients	36	16,155	6470	52.46 (42.96–61.87)	99.30	< 0.001
Northeast China	Health checkups	2	83	10	11.61 (5.31–19.67)	42.60	0.187
	Outpatients	13	5173	1361	52.22 (31.25–72.80)	99.51	< 0.001
Central China	Health checkups	2	579	140	24.15 (20.74–27.73)	0.00	0.370
	Outpatients	35	24,275	10,593	57.31 (48.39–66.01)	99.45	< 0.001
Taiwan	Health checkups	/	/	/	/	/	/
	Outpatients	13	4089	1844	42.15 (24.61–60.78)	99.29	< 0.001
Hong Kong and Macau	Health checkups	/	/	/	/	/	/
	Outpatients	2	858	183	21.28 (18.60–24.10)	0.00	0.431
Multiple regions^a^	Health checkups	/	/	/	/	/	/
	Outpatients	4	1692	929	54.01 (35.53–71.95)	98.15	< 0.001
Sampling site^b^, ^c^							
Head and neck	Health checkups	2	3254	115	3.42 (2.81–4.08)	67.70	0.079
	Outpatients	22	7151	2319	31.94 (16.61–49.54)	99.54	< 0.001
Anal	Health checkups	11	5986	303	5.34 (1.99–9.98)	96.97	< 0.001
	Outpatients	116	109,988	43,922	46.40 (42.54–50.28)	99.39	< 0.001
Genital	Health checkups	3	298	23	8.43 (2.16–17.84)	80.20	0.006
	Outpatients	60	19,359	11,250	66.25 (60.53–71.75)	98.55	< 0.001
Other	Health checkups	/	/	/	/	/	/
	Outpatients	4	371	109	28.33 (17.70–40.30)	82.99	0.0005
Multiple sites	Health checkups	4	5793	115	17.36 (11.48–24.15)	93.80	< 0.001
	Outpatients	59	50,844	25,598	66.15 (60.56–71.52)	99.40	< 0.001
Sampling type							
Exfoliated cells	Health checkups	19	9748	477	7.11 (4.14–10.74)	97.84	< 0.001
	Outpatients	217	132,285	59,938	55.25 (52.20–58.29)	99.39	< 0.001
Biopsies^d^	Health checkups	1	206	3	1.46 (0.50–4.19)	/	/
	Outpatients	37	7453	2329	42.38 (30.55–54.66)	99.18	< 0.001
Tissues^d^	Health checkups	/	/	/	/	/	/
	Outpatients	4	684	554	80.98 (52.30–98.31)	98.06	< 0.001
Sperm	Health checkups	4	1088	95	10.02 (5.74–15.29)	0.00	0.004
	Outpatients	11	3208	776	22.62 (11.85–35.62)	99.01	< 0.001
Serum	Health checkups	1	185	49	26.49 (20.65–33.28)	/	/
	Outpatients	3	3365	1954	41.26 (13.28–72.77)	99.50	< 0.001
Sample size							
< 200	Health checkups	11	1058	130	8.70 (4.27–14.34)	86.57	< 0.001
	Outpatients	103	11,466	7178	64.68 (57.86–71.22)	98.24	< 0.001
≥ 200	Health checkups	13	15,394	1236	7.36 (4.11–11.43)	98.54	< 0.001
	Outpatients	170	181,812	77,172	45.26 (41.90–48.65)	99.53	< 0.001
Whether or not MSM in outpatients^c^							
MSM	HIV positive	23	3443	2399	82.11 (73.66–89.27)	97.00	< 0.001
	HIV negative	19	5693	2958	55.47 (48.79–62.05)	95.66	< 0.001
	HIV unknown	20	5085	2544	53.92 (42.12–65.50)	98.60	< 0.001
Non‐MSM		230	178,514	76,170	50.81 (47.64–53.98)	99.44	< 0.001
Cancer Status in outpatients^c^							
Diagnosed with cancer^e^		26	8195	1790	27.04 (19.37–35.45)	98.38	< 0.001
	HPV 16	11	4428	480	18.50 (9.47–29.64)	98.32	< 0.001
	HPV 18	8	3944	116	5.33 (2.02–10.01)	95.43	< 0.001
	HPV 6	4	2905	12	0.49 (0.04–1.25)	53.47	0.091
	HPV 11	4	2789	26	1.34 (0.30–2.96)	70.72	0.016
No cancer diagnosed		247	184,521	82,201	54.91 (51.92–57.88)	99.40	< 0.001
	HPV 16	175	136,336	8885	7.77 (7.03–8.55)	96.02	< 0.001
	HPV 18	155	123,301	3745	3.53 (3.08–4.00)	93.99	< 0.001
	HPV 6	155	116,111	21,585	19.78 (17.65–22.01)	98.83	< 0.001
	HPV 11	152	116,225	14,263	14.15 (12.45–15.93)	98.58	< 0.001

^a^ Multiple regions referred to the population of one study originate from two or more of the listed regions.

^b^ Oral, esophagus, larynx, hypopharynx, laryngeal, nasal, and auditory canal are classified as head and neck. Anal canal, perianal regions, and rectum are classified as anal. The coronal sulcus, urethra, glans, penis, scrotum, prepuce, prostate gland, and vulvar are classified as genital. Multiple sites indicated that a population was sampled from two or more listed sites. Other sampling sites utilized in this study, which mean one where samples were taken from the bladder and the other where samples were collected from the lung.

^c^ Several studies have reported infection rate results for more than one population or site concurrently, leading to a discrepancy between the number of studies reported and the actual total number of studies conducted.

^d^ Biopsy referred to Formalin‐fixed paraffin‐embedding (FFPE) samples, whereas tissue meant the fresh tissues. The cumulative number of studies across different subgroups does not equal the total number of studies within the grouping, as some studies concurrently report data from multiple subgroups.

^e^ The following cancers were included in this category: oropharyngeal squamous cell carcinoma (OPSCC), Oral squamous cell carcinoma (OSCC), tongue squamous cell carcinoma (TSCC), laryngeal squamous cell carcinoma (LSCC), squamous cell carcinoma of the larynx and hypopharynx (SCCLHP), subungual squamous cell carcinoma (SSCC), esophageal squamous cell carcinoma (ESCC), lung SCC, penile cancer, bladder cancer, gastric cancer, and prostatic carcinoma. HPV, human papillomavirus; MSM, men who have sex with men.

Among outpatients stratified by MSM status, HPV prevalence is the highest among MSM who were HIV positive (82.11%; 95% CI, 73.66–89.27). Predominant HR genotypes include HPV 16 (12.67%; 95% CI, 10.58–14.90 in MSM; 7.35%; 95% CI, 6.54–8.20 in non‐MSM) and HPV 52 (8.08%; 95% CI, 6.46–9.85 in MSM; 3.95%; 95% CI, 3.48–4.45 in non‐MSM) (Appendix S1). LR genotypes HPV 6 and 11 are most prevalent in both MSM (20.57% and 14.28%, respectively) and non‐MSM (18.92% and 13.43%, respectively) populations (Appendix S1). Among outpatients diagnosed with cancer, the overall HPV prevalence is 27.04%, with HPV 16 (18.50%; 95% CI, 9.47–29.64) and 18 (5.33%; 95% CI, 2.02–10.01) as the predominant subtypes compared with HPV 6 (0.49%; 95% CI, 0.04–1.25) and 11 (1.34%; 95% CI, 0.30–2.96) (Table 2).

### 3.3 Age Distribution and HPV Positivity Among Chinese Male Outpatients and Health Checkups

**FIGURE 3:**
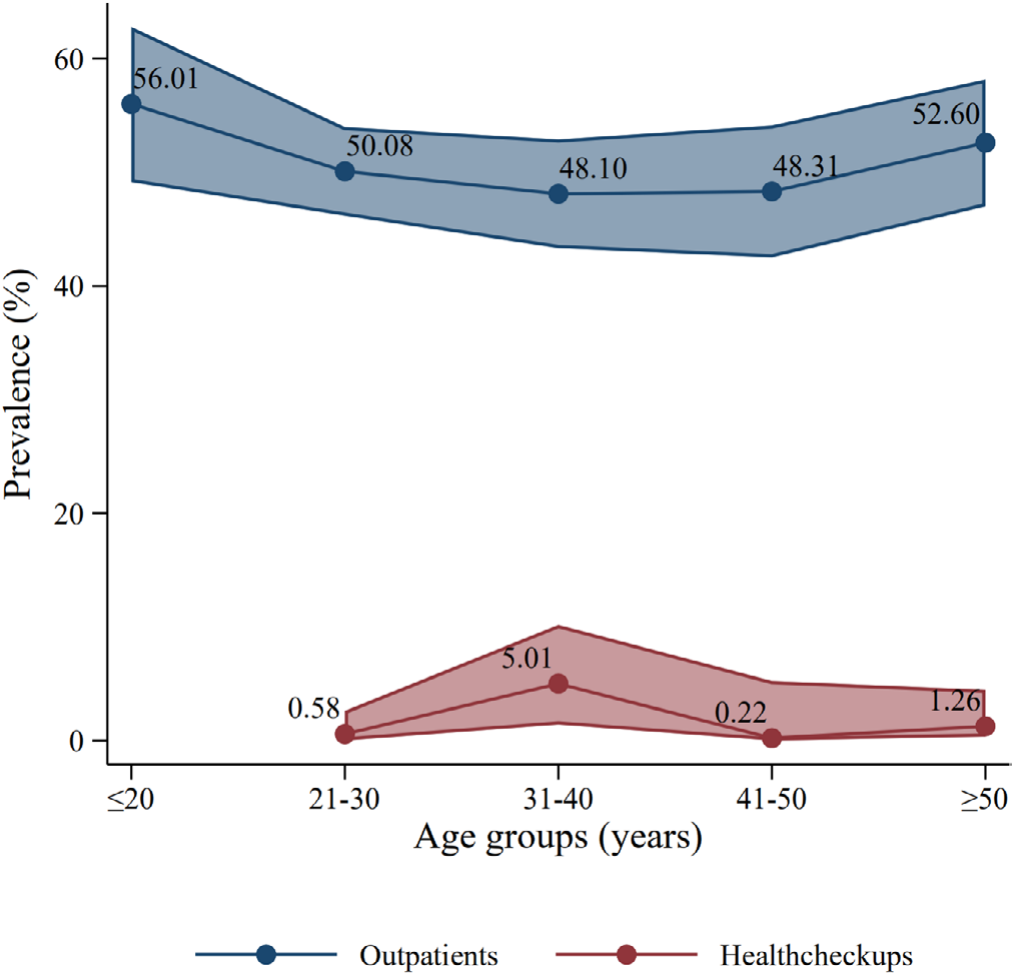
Age distribution among male HPV infections in China.

The predominant age group of outpatients included in this study was between 21 and 40 (69.00%, 43,793/63,470), and a small proportion (4.37%, 2771/63,470) were under the age of 20 (Appendix S1). HPV positivity rates remained high across age groups, ranging from 48.10% in the 31–40 age group to 56.01% in the age group of 20 or younger among outpatients seeking medical assistance (Figure 3). Among health checkups, the predominant age group is 31–40 years (51.08%, 475/930), with a prevalence rate of 5.01% (95% CI, 1.42–10.18) (Appendix S1).

## 4 Discussion

This comprehensive meta‐analysis, including data from 215,685 HPV‐tested males across 296 studies, significantly enhances our understanding of HPV prevalence among Chinese men. To our knowledge, this study is the first to assess both overall HPV positivity and genotype distribution within the male population in China. Our findings illuminate the substantial extent of HPV infection among Chinese men, underscoring the necessity of utilizing existing prophylactic HPV vaccines to mitigate HPV‐associated diseases in this demographic.

Among males undergoing health checkups, which better represent the general male population, the overall HPV positivity was 7.89%. This figure is notably lower than the 15% reported for males in Eastern and South‐Eastern Asia [18] and the global prevalence of 31.0% among men [18]. Also, it was much lower than the 21.6% positivity observed in Chinese females undergoing routine cervical cancer screening [19]. Among HPV‐positive health checkup males, the most common HPV genotypes were HPV 16 (3.66%), 52 (1.37%), 58 (1.19%), 6 (1.03%), and 18 (1.00%), aligning with global trends in male populations [18, 20]. The predominance of single HR‐HPV infections, particularly HPV 16, 52, and 58, reflects patterns seen in the general female population [19, 21, 22] and emphasizes the importance of addressing men's role in HPV transmission. These consistent trends highlight the urgent need for comprehensive prevention strategies that target both genders, as HPV genotypes are detected across populations [23].

In outpatients, the overall HPV positivity rate of 52.45% aligns closely with the global estimates of male HPV infection, which average around 49.0% [24]. Notably, this rate surpasses that documented among Vietnamese male patients (20.3%) [25]. Our findings revealed that single‐type infections (30.82%) were more common than multiple infections (21.40%), with single LR‐HPV infection (19.75%) being the most prevalent. Among the outpatients, HPV 16 (8.29%), 52 (4.64%) and 58 (3.71%) emerged as the top HR‐HPV genotypes, whereas HPV 6 (19.06%), and 11 (13.71%) were the most common LR‐HPV genotypes. These distributions resonate with studies from Vietnamese and Iranian male patients [25, 26], reinforcing the need for targeted interventions.

The observed lower prevalence of HPV among health checkup males compared to outpatients can be attributed to several factors. Health checkups are more representative of the general male population, many of whom may be asymptomatic and unaware of their HPV status, as many HPV infections do not manifest symptoms, particularly those caused by low‐risk types. Studies have shown that HPV infections can remain latent and asymptomatic, with many individuals clearing the virus naturally without clinical intervention [27]. In contrast, outpatients typically seek medical care when symptomatic, such as when presenting with genital warts or lesions associated with high‐risk HPV types, leading to a higher observed prevalence among this group. Outpatients may also include high‐risk groups such as MSM, individuals with immunosuppressive conditions (e.g., HIV‐positive patients), and those with HPV‐related cancers, all of whom are more susceptible to HPV infections [28]. Moreover, health checkups often do not include routine HPV testing, which may further contribute to the lower detection rates in this group. In global comparisons, HPV prevalence in men has been reported to vary, with higher rates found in high‐risk groups such as MSM [29]. This suggests that the higher HPV prevalence among outpatients likely reflects their higher likelihood of exhibiting clinical symptoms and seeking care for HPV‐related conditions, whereas health checkup populations include a broader spectrum of individuals, many of whom may be asymptomatic carriers of the virus. Comparing HPV prevalence data across countries is essential to understand the global landscape and the role of specific risk factors in different populations.

It is well‐documented that HPV infection is linked to several cancers, including head and neck squamous cell carcinoma (HNSCC), penile cancer, and anal cancer [30, 31]. Our analysis indicated that among outpatients diagnosed with cancer, HPV 16 (18.50%) and 18 (5.33%) were the most prevalent genotypes. In contrast, HPV 6 (19.78%) and 11 (14.15%) dominated among outpatients not diagnosed with cancer. This suggests that HPV 16 and 18 are strongly associated with cancer development in Chinese males, paralleling findings in females [32]. Furthermore, HPV 6 and 11, which are more commonly linked to anogenital warts, represent a significant HPV‐related disease burden in men [33]. Our results underscore that the majority of prevalent genotypes are covered by HPV vaccines, which have demonstrated effectiveness against HPV infection and related diseases globally [34, 35]. Future research is needed to evaluate the effectiveness of these vaccines in preventing HPV‐related diseases among Chinese males.

The prevalence of HPV infection is particularly high among MSM in China, revealing a stark contrast between HIV‐positive and HIV‐negative individuals. The HPV prevalence among HIV‐positive MSM was 82.11%, consistent with previous meta‐analysis, indicating a prevalence of 85.1% in this group [29]. In comparison, HIV‐negative MSM showed a prevalence of 55.47%, akin to heterosexual males (50.91%) [36]. The most frequently detected HPV types among MSM included HR‐HPV 16 (12.67%), 52 (8.08%), 18 (7.30%), and LR‐HPV 6 (20.57%), 11 (14.28%). Given that current vaccines target these prevalent types and have shown efficacy, implementing targeted vaccination programs for MSM could significantly reduce infection rates and the burden of HPV‐related diseases in this high‐risk population.

Geographic disparities in HPV infection rates among Chinese males were also evident, with central China exhibiting higher HPV prevalence (24.15% in health checkups, 57.31% in outpatients) compared to Eastern China (4.92% in health checkups, 52.48% in outpatients). Notably, Hong Kong and Macau reported the lowest rates at 21.28%, likely due to earlier vaccine availability for both genders in these regions [37]. These findings highlight the potential impact of gender‐neutral vaccination programs in curbing HPV‐related diseases and lowering HPV infection rates among males. Sampling sites and methods further influenced HPV prevalence rates, with tissue samples revealing the highest prevalence (80.98%), whereas sperm samples yielded the lowest rates. The performance of HPV 16 in sperm samples raises concerns about potential links to male infertility, particularly regarding sperm motility and morphology [38, 39]. This suggests that HPV infection poses broader implications beyond cancer, emphasizing the need for vaccination to preserve male fertility.

This study has several strengths, notably its robust estimate of HPV prevalence among Chinese males derived from a substantial number of high‐quality studies. The analysis encompassed various parameters, including outpatients, health checkups, geographic region, sampling site, sampling type, and age group, and investigated HPV infection in MSM and cancer‐diagnosed males, providing significant insights for future vaccination strategies. However, limitations must be acknowledged. A high degree of heterogeneity was observed between studies, which subgroup analyses and meta‐regression could not fully elucidate. Additionally, variability in HPV genotype detection methods across studies may have influenced the reported prevalence, yet the large sample size mitigates concerns about significant bias. Furthermore, publication bias was detected among outpatients, despite visual inspection suggesting symmetry in funnel plots. Finally, the age distribution of HPV prevalence among outpatients may be skewed, as certain age groups are more likely to seek medical assistance when symptomatic, potentially underrepresenting the true prevalence of HPV infection.

In conclusion, this study reveals a high prevalence of HPV infection among Chinese males, particularly in outpatients and among MSM. The most prevalent genotypes—HPV16, 18, 52, 58, 6, and 11—underscore the critical need for targeted vaccination strategies. Implementing gender‐neutral HPV vaccination programs could substantially reduce the burden of HPV infection and HPV‐related diseases across genders, informing future public health efforts in China and similar regions and countries.

## Author Contributions

J.L. conceptualized the study. Y.L. designed the methodology and conducted the literature searches alongside F.Z. Data curation was performed by C.Q., F.Z., Y.L., Y.Y., H.W., C.L., S.Q., W.J., C.L., Y.Y., X.G., H.R., N.J., N.L., and Y.L. Y.L. and C.Q. drafted the original manuscript, with all authors contributing to the review and editing. The study was supervised by D.W. and J.L.

## Ethics Statement

The authors have nothing to report.

## Conflicts of Interest

The authors declare no conflicts of interest.

## Supporting information


Appendix S1.

## Data Availability

The data supporting this article are included within the article itself and in the Supporting Information.
