# Supplementary material for: Prevalence of Human Papillomavirus and Genotype Distribution in Chinese Men: A Systematic Review and Meta‐Analysis
Source: Cancer Med. 2025 Feb 17;14(4):e70686. doi: 10.1002/cam4.70686 (PMC11831462; doi:10.1002/cam4.70686)
Supplement: Supplementary file 1 — Appendix S1. [file CAM4-14-e70686-s001.docx]

***Appendixes***

[***Appendix1: Search strategy*** 1](#_Toc186640151)

[***Appendix 2 The information extracted in this meta-analysis*** 2](#_Toc186640152)

[***Appendix 3: Quality assessment of original studies*** 3](#_Toc186640153)

[***Appendix4: Meta-regression results*** 10](#_Toc186640154)

[***Appendix5: Characteristics of studies examining the prevalence of HPV positivity among Chinese males*** 10](#_Toc186640155)

[***Appendix6: Results of funnel plot, egger’s test, and trim and fill analysis*** 23](#_Toc186640156)

[***Appendix7: Pooled prevalence of specific HPV genotypes among MSM and non-MSM*** 25](#_Toc186640157)

[***Appendix 8: Prevalence of any HPV among males in different age groups in China, by population group*** 26](#_Toc186640158)

[***Appendix 9: Sensitivity analysis results*** 26](#_Toc186640159)

***Appendix1: Search strategy***

| Database | Keywords | Search strategy | Numbers |
| --- | --- | --- | --- |
| WOS core collection | China; Chinese; Taiwan; HongKong; Macau; men; male; HPV; Human Papillomavirus; Papillomaviridae | #1 (((TS=(HPV)) OR TS=(papillomaviridae)) OR TS=(human papilloma virus)) OR TS=(human papillomavirus)  #2 (TS=(men)) OR TS=(male)  #3 ((((TS=(China)) OR TS=(Chinese)) OR TS=(Taiwan)) OR TS=(Hongkong)) OR TS=(Macau)  #4 #1 AND #2 AND #3  Time: 2012-01-01 to 2024-09-25 | 340 |
| CNKI | 男性；HPV；人乳头瘤病毒 | (主题=男性) AND (主题=( HPV + 人乳头瘤病毒 + 人乳头状瘤病毒))  Time: 2012-01-01 to 2024-09-25 | 442 |
| VIP | 男性；HPV；人乳头瘤病毒 | (((题名或关键词=男性 AND ((题名或关键词=HPV OR 题名或关键词=人乳头瘤病毒) OR 题名或关键词=人乳头状瘤病毒)))) AND (years:[2012 TO 2023])Time: 2012-01-01 to 2024-09-25 | 232 |
| Medline | China; Chinese; Taiwan; HongKong; Macau; men; male; HPV; Human Papillomavirus; Papillomaviridae | #1 (((TS=(HPV)) OR TS=(papillomaviridae)) OR TS=(human papilloma virus)) OR TS=(human papillomavirus)  #2 (TS=(men)) OR TS=(male)  #3 ((((TS=(China)) OR TS=(Chinese)) OR TS=(Taiwan)) OR TS=(Hongkong)) OR TS=(Macau)  #4 #1 AND #2 AND #3  Time: 2012-01-01 to 2024-09-25 | 415 |
| Wanfang | 男性；HPV；人乳头瘤病毒 | (主题:(男性) and 主题:(HPV OR 人乳头瘤病毒 OR 人乳头状瘤病毒)) and 发表时间:2012-2023Time: 2012-01-01 to 2024-09-25 | 748 |
| SinoMed | 男性；HPV；人乳头瘤病毒 | ((("HPV"[常用字段:智能] OR "人乳头瘤病毒"[常用字段:智能] OR "人乳头状瘤病毒"[常用字段:智能]) AND (("男性"[常用字段:智能]))) AND 2012-2023[日期])  Time: 2012-01-01 to 2023-12-31Time: 2012-01-01 to 2024-09-25 | 492 |
| Embase(Ovid) | China; Chinese; Taiwan; HongKong; Macau; men; male; HPV; Human Papillomavirus; Papillomaviridae | #1 (HPV or papiliomaviridae or human papilloma virus or human papillomavirus).mp. [mp=title, abstract, heading word, drug trade name, original title, device manufacturer, drug manufacturer, device trade name, keyword heading word, floating subheading word, candidate term word]  #2 (men or male).mp. [mp=title, abstract, heading word, drug trade name, original title, device manufacturer, drug manufacturer, device trade name, keyword heading word, floating subheading word, candidate term word]  #3 (China or Chinese or Taiwan or Hongkong or Macau).mp. [mp=title, abstract, heading word, drug trade name, original title, device manufacturer, drug manufacturer, device trade name, keyword heading word, floating subheading word, candidate term word]  #4 1 and 2 and 3  #5 limit 4 to yr="2012 - 2024" | 698 |
| Cochrane library | China; Chinese; Taiwan; HongKong; Macau; men; male; HPV; Human Papillomavirus; Papillomaviridae | ID Search Hits  #1 MeSH descriptor: [Papillomaviridae] explode all trees 954  #2 Human Papilloma Virus or Human Papilloma Viruses or Human Papillomavirus Viruses or Human Papillomavirus Virus or Human Papillomavirus or HPV or Papillomaviridae 4442  #3 #1 or #2 4444  #4 China or Chinese or Taiwan or Hong kong or Macau 128720  #5 male or men 903049  #6 #3 and #4 and #5 with Cochrane Library publication date Between Jan 2012 and Sep 2024, in Trials (Word variations have been searched) 38 | 42 |
| Total |  |  | 3409 |

***Appendix 2 The information extracted in this meta-analysis***

| Item | Description | Item | Description |
| --- | --- | --- | --- |
| paper identification | the first author’s name; publication year | the anatomical site | head and neck; genital; anal, etc. |
| region of the study | Eastern China, Western China, etc. | tissue type | exfoliated cells; biopsies; tissues; sperm, etc. |
| participants description | age range, diseases, etc. | laboratory methods for HPV diagnosis | Polymerase Chain Reaction (PCR); others |
| study design | cross-sectional study; case control study, or others | PCR primers and HPV typing method | Reverse Dot Blot, FQ-PCR, etc. |
| sample size | NA | The number of males tested / The number of HPV infections | any genotype, high-risk (HR) genotype, low-risk (LR) genotype, multiple genotypes*, single genotype, specified age groups, etc. |
| data collection period | NA | HPV genotypes | HR-HPV: 16, 18, 31, 33, 35, 39, 45, 51, 52, 56, 58, 59; LR-HPV: 6, 11, 26, 34, 40, 42, 43, 44, 53, 54, 55, 61, 66-73, 81-84; |
| ... | ... |  |  |

*Multiple genotype infection is defined as a simultaneous infection of more than one HPV genotype

***Appendix 3: Quality assessment of original studies***

|  | **Quality Assessment Score*** | | | | | | | | | | | | |
| --- | --- | --- | --- | --- | --- | --- | --- | --- | --- | --- | --- | --- | --- |
|  | **Study** | **Q1** | **Q2** | **Q3** | **Q4** | **Q5** | **Q6** | **Q7** | **Q8** | **Q9** | **Q10** | **Q11** | **Total** |
| 1 | Cao F et al.2014 | Y | Y | Y | Y | Y | Y | Y | Y | Y | Y | N | 10 |
| 2 | Chen L et al.2021 | Y | Y | Y | Y | Y | Y | Y | Y | Y | Y | N | 10 |
| 3 | Chen X et al.2016 | Y | N | N | Y | Y | Y | Y | N | Y | Y | N | 7 |
| 4 | Cheng S et al.2012 | Y | Y | Y | Y | Y | Y | Y | Y | Y | Y | N | 10 |
| 5 | Cheng S et al.2018 | Y | Y | Y | Y | Y | Y | Y | N | Y | Y | N | 9 |
| 6 | Cheng Y et al.2012 | Y | Y | Y | Y | Y | Y | Y | N | Y | Y | N | 9 |
| 7 | Chu C et al.2020 | Y | Y | Y | U | Y | Y | Y | Y | Y | Y | Y | 10 |
| 8 | Cong X et al.2016 | Y | Y | Y | Y | Y | Y | Y | Y | Y | Y | N | 10 |
| 9 | Cui L et al.2019 | Y | Y | Y | Y | Y | Y | Y | Y | Y | Y | N | 10 |
| 10 | Fan S et al.2020 | Y | Y | Y | Y | Y | Y | N | Y | Y | N | N | 8 |
| 11 | Gu W et al.2020 | Y | Y | Y | U | Y | Y | Y | Y | Y | Y | N | 9 |
| 12 | He ZH et al.2013 | Y | Y | Y | Y | Y | Y | Y | Y | Y | Y | N | 10 |
| 13 | Hu JM et al.2013 | Y | Y | Y | Y | Y | Y | Y | Y | Y | Y | N | 10 |
| 14 | Hu Y et al.2013 | Y | Y | Y | Y | Y | Y | Y | Y | Y | Y | N | 10 |
| 15 | Lee L et al.2015 | Y | Y | Y | Y | Y | Y | N | Y | Y | Y | Y | 10 |
| 16 | Li P et al.2021 | Y | Y | Y | Y | Y | Y | Y | Y | Y | Y | N | 10 |
| 17 | Li S et al.2017 | Y | Y | Y | Y | Y | Y | Y | N | Y | Y | N | 9 |
| 18 | Li W et al.2017 | Y | Y | Y | Y | Y | Y | Y | Y | Y | Y | N | 10 |
| 19 | Li X et al.2016 | Y | Y | Y | Y | Y | Y | Y | Y | Y | Y | N | 10 |
| 20 | Li X et al.2021 | Y | Y | Y | Y | Y | Y | Y | Y | Y | Y | N | 10 |
| 21 | Li Y et al.2021 | Y | Y | Y | Y | Y | Y | Y | Y | Y | Y | N | 10 |
| 22 | Li Z et al.2015 | Y | Y | Y | Y | Y | Y | Y | Y | Y | Y | N | 10 |
| 23 | Lin C et al.2018 | Y | Y | Y | Y | Y | Y | Y | Y | Y | Y | N | 10 |
| 24 | Liu T et al.2013 | Y | Y | Y | Y | Y | Y | Y | Y | Y | Y | N | 10 |
| 25 | Liu X et al.2019 | Y | Y | Y | Y | Y | Y | N | Y | Y | Y | N | 9 |
| 26 | Luo Z et al.2015 | Y | Y | Y | Y | Y | Y | Y | N | Y | Y | N | 9 |
| 27 | Ma D et al.2019 | Y | Y | Y | Y | Y | Y | N | Y | Y | Y | N | 9 |
| 28 | Meng H et al.2018 | Y | Y | Y | Y | Y | Y | Y | Y | Y | Y | N | 10 |
| 29 | Mujtaba H et al.2018 | Y | Y | Y | Y | Y | Y | Y | Y | Y | Y | N | 10 |
| 30 | Ni Y et al.2022 | Y | Y | Y | Y | Y | Y | Y | Y | Y | Y | N | 10 |
| 31 | Ning H et al.2015 | Y | Y | Y | Y | Y | Y | Y | Y | Y | Y | N | 10 |
| 32 | Peng J et al.2013 | Y | Y | Y | Y | Y | Y | Y | Y | Y | Y | N | 10 |
| 33 | Peng J et al.2016 | Y | Y | Y | Y | Y | Y | Y | Y | Y | Y | N | 10 |
| 34 | Qian, H et al.2017 | Y | Y | N | Y | Y | Y | Y | N | Y | Y | N | 8 |
| 35 | Ren X et al.2017 | Y | Y | Y | Y | Y | Y | Y | Y | Y | Y | N | 10 |
| 36 | Strong C et al.2020 | Y | Y | Y | Y | Y | Y | Y | Y | Y | Y | N | 10 |
| 37 | Su Y et al.2019 | Y | Y | Y | Y | Y | Y | Y | Y | Y | Y | N | 10 |
| 38 | Tong F et al.2018 | Y | Y | N | Y | Y | Y | Y | Y | Y | Y | N | 9 |
| 39 | Wang C et al.2019 | Y | Y | Y | Y | Y | Y | Y | Y | Y | Y | N | 10 |
| 40 | Wang H et al.2021 | Y | Y | Y | Y | Y | Y | Y | Y | Y | Y | N | 10 |
| 41 | Wang Y et al.2013 | Y | Y | Y | Y | Y | Y | Y | Y | Y | Y | N | 10 |
| 42 | Wei F et al.2016 | Y | Y | Y | Y | Y | Y | N | Y | Y | Y | N | 9 |
| 43 | Wong M et al.2018 | Y | Y | N | Y | Y | Y | Y | Y | Y | Y | N | 9 |
| 44 | Wu P et al.2020 | Y | Y | Y | U | Y | Y | Y | Y | Y | Y | N | 9 |
| 45 | Xiang J et al.2021 | Y | Y | Y | Y | Y | Y | Y | Y | Y | Y | N | 10 |
| 46 | Xin H et al.2017 | Y | Y | Y | Y | Y | Y | Y | Y | Y | Y | N | 10 |
| 47 | Xu T et al.2020 | Y | Y | Y | Y | Y | Y | N | N | Y | Y | N | 8 |
| 48 | Xu Y et al.2014 | Y | Y | N | U | Y | Y | Y | N | Y | Y | N | 7 |
| 49 | Xu Y et al.2015 | Y | Y | Y | Y | Y | Y | Y | N | Y | Y | N | 9 |
| 50 | Xu Y et al.2021 | Y | Y | Y | Y | Y | Y | Y | Y | Y | Y | N | 10 |
| 51 | Yan Y et al.2021 | Y | Y | Y | Y | Y | Y | Y | Y | Y | Y | N | 10 |
| 52 | Yang Y et al.2013 | Y | Y | N | Y | Y | Y | Y | N | Y | Y | N | 8 |
| 53 | Yang Y et al.2012 | Y | Y | Y | Y | Y | Y | Y | Y | Y | Y | N | 10 |
| 54 | Ye Z et al.2021 | Y | Y | Y | Y | Y | Y | Y | Y | Y | Y | N | 10 |
| 55 | Yin W et al.2020 | Y | Y | Y | Y | Y | Y | Y | Y | Y | Y | N | 10 |
| 56 | Yu C et al.2013 | Y | Y | Y | Y | Y | Y | Y | Y | Y | Y | N | 10 |
| 57 | Yu Y et al.2013 | Y | Y | Y | Y | Y | Y | Y | N | Y | Y | N | 9 |
| 58 | Zhang Y et al.2017 | Y | Y | Y | Y | Y | Y | Y | Y | Y | Y | N | 10 |
| 59 | Zhang C et al.2018 | Y | Y | Y | Y | Y | Y | Y | Y | Y | Y | N | 10 |
| 60 | Zhang C et al.2017 | Y | Y | Y | Y | Y | Y | Y | Y | Y | Y | Y | 11 |
| 61 | Zhang C et al.2015 | Y | Y | Y | Y | Y | Y | Y | Y | Y | Y | N | 10 |
| 62 | Zhang D et al.2014 | Y | Y | Y | Y | Y | Y | Y | Y | Y | Y | N | 10 |
| 63 | Zhang J et al.2022 | Y | Y | Y | Y | Y | Y | Y | Y | Y | Y | N | 10 |
| 64 | Zhang X et al.2013 | Y | Y | Y | Y | Y | Y | Y | Y | Y | Y | N | 10 |
| 65 | Zhong Y et al.2019 | Y | Y | Y | Y | Y | Y | N | Y | Y | Y | N | 9 |
| 66 | Zhou Y et al.2022 | Y | Y | Y | Y | Y | Y | Y | Y | Y | Y | N | 10 |
| 67 | Zhou Y et al.2020 | Y | Y | Y | Y | Y | Y | Y | Y | Y | Y | N | 10 |
| 68 | Chang Y et al.2021 | Y | Y | Y | Y | Y | Y | N | Y | Y | Y | N | 9 |
| 69 | Chen T et al.2018 | Y | Y | Y | Y | Y | Y | Y | Y | Y | Y | N | 10 |
| 70 | Chen W et al.2016 | Y | Y | Y | Y | Y | Y | Y | N | Y | Y | N | 9 |
| 71 | Chen Y et al.2014 | Y | Y | Y | Y | Y | Y | N | Y | Y | Y | N | 9 |
| 72 | Chen Z et al.2016 | Y | Y | Y | Y | Y | Y | Y | Y | Y | Y | N | 10 |
| 73 | Deng D et al.2019 | Y | Y | Y | Y | Y | Y | Y | N | Y | Y | N | 9 |
| 74 | Deng G et al.2016 | Y | Y | N | Y | Y | Y | Y | N | Y | Y | N | 8 |
| 75 | Deng J et al.2014 | Y | Y | Y | Y | Y | Y | Y | Y | Y | Y | N | 10 |
| 76 | Dong z et al.2016 | Y | Y | Y | Y | Y | Y | Y | Y | Y | Y | N | 10 |
| 77 | Fang B et al.2018 | Y | Y | Y | Y | Y | Y | Y | Y | Y | Y | N | 10 |
| 78 | Feng L et al.2019 | Y | Y | Y | Y | Y | Y | Y | Y | Y | Y | N | 10 |
| 79 | Gao M et al.2019 | Y | Y | Y | Y | Y | Y | Y | Y | Y | Y | N | 10 |
| 80 | Guo S et al.2018 | Y | Y | Y | Y | Y | Y | N | Y | Y | Y | N | 9 |
| 81 | Hao G et al.2022 | Y | Y | Y | Y | Y | Y | N | Y | Y | Y | N | 9 |
| 82 | Huang J et al.2015 | Y | Y | Y | Y | Y | Y | Y | Y | Y | Y | N | 10 |
| 83 | Huang Y et al.2015 | Y | Y | Y | Y | Y | Y | Y | N | Y | Y | N | 9 |
| 84 | Huang Z et al.2013 | Y | Y | Y | Y | Y | Y | Y | Y | Y | Y | N | 10 |
| 85 | Xi M et al.2022 | Y | Y | Y | Y | Y | Y | Y | Y | Y | Y | N | 10 |
| 86 | Zhao M et al.2013 | Y | Y | Y | Y | Y | Y | Y | N | Y | Y | N | 9 |
| 87 | Fu Y et al.2018 | Y | Y | Y | Y | Y | Y | Y | Y | Y | Y | N | 10 |
| 88 | Gao M et al.2019 | Y | Y | Y | Y | Y | Y | Y | Y | Y | Y | N | 10 |
| 89 | Li T et al.2012 | Y | Y | Y | Y | Y | Y | Y | Y | Y | Y | N | 10 |
| 90 | Li Y et al.2017 | Y | Y | Y | Y | Y | Y | Y | Y | Y | Y | N | 10 |
| 91 | Liang H et al.2013 | Y | Y | Y | Y | Y | Y | Y | Y | Y | Y | N | 10 |
| 92 | Liang X et al.2014 | Y | Y | Y | Y | Y | Y | Y | Y | Y | Y | N | 10 |
| 93 | Lin Q et al.2013 | Y | Y | Y | Y | Y | Y | Y | Y | Y | Y | N | 10 |
| 94 | Min X et al.2017 | Y | Y | Y | Y | Y | Y | Y | Y | Y | Y | N | 10 |
| 95 | Pu J et al.2013 | Y | Y | Y | Y | Y | Y | Y | Y | Y | Y | N | 10 |
| 96 | Sun X et al.2014 | Y | Y | Y | Y | Y | Y | Y | Y | Y | Y | N | 10 |
| 97 | Tian T et al.2017 | Y | Y | Y | Y | Y | Y | Y | Y | Y | Y | N | 10 |
| 98 | Wang W et al.2013 | Y | Y | Y | Y | Y | Y | Y | N | Y | Y | N | 9 |
| 99 | Wang Z et al.2014 | Y | Y | Y | Y | Y | Y | Y | Y | Y | Y | N | 10 |
| 100 | Wu Y et al.2020 | Y | Y | Y | Y | Y | Y | Y | Y | Y | Y | N | 10 |
| 101 | Xu Y et al.2017 | Y | Y | Y | Y | Y | Y | Y | Y | Y | Y | N | 10 |
| 102 | Yang A et al.2018 | Y | Y | Y | Y | Y | Y | Y | U | Y | Y | N | 9 |
| 103 | Yang Q et al.2019 | Y | Y | N | Y | Y | Y | Y | Y | Y | Y | N | 9 |
| 104 | Yao Y et al.2021 | Y | Y | Y | Y | Y | Y | Y | N | Y | Y | N | 9 |
| 105 | Zhong W et al.2021 | Y | Y | Y | Y | Y | Y | Y | Y | Y | Y | N | 10 |
| 106 | Zhong M et al.2016 | Y | Y | Y | Y | Y | Y | Y | Y | Y | Y | N | 10 |
| 107 | Zhu Z et al.2018 | Y | Y | Y | Y | Y | Y | Y | Y | Y | Y | N | 10 |
| 108 | Luo J et al.2019 | Y | Y | Y | Y | Y | Y | Y | U | Y | Y | N | 9 |
| 109 | Mo H et al.2015 | Y | Y | Y | Y | Y | Y | Y | Y | Y | Y | N | 10 |
| 110 | Muergan M et al.2013 | Y | Y | Y | Y | Y | Y | Y | N | Y | Y | N | 9 |
| 111 | Pan L et al.2018 | Y | Y | Y | Y | Y | Y | N | Y | Y | Y | N | 9 |
| 112 | Qiao Y et al.2020 | Y | Y | Y | Y | Y | Y | N | N | Y | Y | N | 8 |
| 113 | Ruan J et al.2012 | Y | Y | Y | Y | Y | Y | Y | Y | Y | Y | N | 10 |
| 114 | Shen X et al.2013 | Y | Y | Y | Y | Y | Y | N | Y | Y | Y | N | 9 |
| 115 | Sun W et al.2014 | Y | Y | Y | Y | Y | Y | N | N | Y | Y | Y | 9 |
| 116 | Tuo Y et al.2015 | Y | Y | Y | Y | Y | Y | Y | N | Y | Y | N | 9 |
| 117 | Wan C et al.2018 | Y | Y | Y | Y | Y | Y | Y | N | Y | Y | N | 9 |
| 118 | Wang M et al.2015 | Y | Y | Y | Y | Y | Y | Y | Y | Y | Y | N | 10 |
| 119 | Wang C et al.2013 | Y | Y | Y | Y | Y | Y | Y | Y | Y | U | N | 9 |
| 120 | Qi C et al.2017 | Y | Y | Y | Y | Y | Y | N | Y | Y | Y | N | 9 |
| 121 | Wang G et al.2020 | Y | Y | Y | Y | Y | Y | Y | Y | Y | Y | N | 10 |
| 122 | Wei J et al.2022 | Y | Y | Y | Y | Y | Y | Y | Y | Y | Y | N | 10 |
| 123 | Wu Y et al.2015 | Y | Y | Y | Y | Y | Y | Y | N | Y | Y | N | 9 |
| 124 | Yao X et al.2015 | Y | Y | Y | Y | Y | Y | Y | Y | Y | Y | N | 10 |
| 125 | Yu G et al.2018 | Y | Y | Y | Y | Y | Y | Y | Y | Y | Y | N | 10 |
| 126 | Yu J et al.2016 | Y | Y | Y | Y | Y | Y | Y | Y | Y | Y | N | 10 |
| 127 | Zhang G et al.2015 | Y | Y | N | U | Y | Y | Y | N | Y | Y | N | 7 |
| 128 | Zhang J et al.2017 | Y | Y | Y | U | Y | Y | Y | Y | Y | Y | N | 9 |
| 129 | Zhang R et al.2018 | Y | Y | N | U | Y | Y | N | Y | Y | Y | N | 7 |
| 130 | Zhang R et al.2017 | Y | Y | Y | Y | Y | Y | Y | Y | Y | Y | N | 10 |
| 131 | Zhao X et al.2017 | Y | Y | Y | Y | Y | Y | N | Y | Y | Y | N | 9 |
| 132 | Zheng Y et al.2021 | Y | Y | Y | Y | Y | Y | N | Y | Y | Y | N | 9 |
| 133 | Chen J et al.2020 | Y | Y | Y | Y | Y | Y | Y | Y | Y | Y | N | 10 |
| 134 | Cheng Y et al.2017 | Y | Y | Y | Y | Y | Y | Y | N | Y | Y | N | 9 |
| 135 | Cheng L et al.2021 | Y | Y | Y | Y | Y | Y | Y | Y | Y | Y | N | 10 |
| 136 | Duan B et al.2021 | Y | Y | Y | Y | Y | Y | Y | Y | Y | Y | N | 10 |
| 137 | Fan J et al.2018 | Y | Y | Y | Y | Y | Y | Y | N | Y | Y | Y | 10 |
| 138 | Fang W et al.2013 | Y | Y | Y | Y | Y | Y | Y | Y | Y | Y | N | 10 |
| 139 | Fu Y et al.2020 | Y | Y | Y | Y | Y | Y | Y | Y | Y | Y | N | 10 |
| 140 | Gao Y et al.2019 | Y | Y | Y | Y | Y | Y | Y | Y | Y | Y | N | 10 |
| 141 | Han w et al.2017 | Y | Y | Y | Y | Y | Y | Y | Y | Y | Y | N | 10 |
| 142 | He Y et al.2015 | Y | Y | Y | Y | Y | Y | Y | Y | Y | Y | N | 10 |
| 143 | Hu F et al.2014 | Y | Y | Y | Y | Y | Y | Y | Y | Y | Y | N | 10 |
| 144 | Huang Z et al.2018 | Y | Y | Y | Y | Y | Y | Y | Y | Y | Y | N | 10 |
| 145 | Ji Z et al.2022 | Y | Y | Y | Y | Y | Y | Y | Y | Y | Y | N | 10 |
| 146 | Jiang L et al.2018 | Y | Y | Y | Y | Y | Y | Y | Y | Y | Y | N | 10 |
| 147 | Li J et al.2014 | Y | Y | Y | Y | Y | Y | Y | Y | Y | Y | N | 10 |
| 148 | Li L et al.2012 | Y | Y | Y | Y | Y | Y | Y | N | Y | Y | Y | 10 |
| 149 | Liang M et al.2022 | Y | Y | Y | Y | Y | Y | Y | Y | Y | Y | N | 10 |
| 150 | Liao N et al.2013 | Y | Y | Y | Y | Y | Y | Y | Y | Y | Y | N | 10 |
| 151 | Liu M et al.2014 | Y | Y | Y | Y | Y | Y | Y | Y | Y | Y | N | 10 |
| 152 | Zhao Q et al.2016 | Y | N | Y | Y | Y | Y | Y | Y | Y | Y | N | 9 |
| 153 | Zhao X et al.2017 | Y | Y | Y | Y | Y | Y | Y | Y | Y | Y | N | 10 |
| 154 | Zhao X et al.2016 | Y | Y | Y | Y | Y | Y | Y | N | Y | Y | N | 9 |
| 155 | Zhong Y et al.2018 | Y | Y | Y | Y | Y | Y | Y | Y | Y | Y | N | 10 |
| 156 | Zhu K et al.2018 | Y | N | Y | Y | Y | Y | Y | N | Y | Y | N | 8 |
| 157 | Wang G et al.2019 | Y | Y | Y | Y | Y | Y | U | Y | Y | Y | N | 9 |
| 158 | Wang J et al.2014 | Y | Y | Y | Y | Y | Y | Y | Y | Y | Y | N | 10 |
| 159 | Wu M et al.2017 | Y | Y | Y | Y | Y | Y | Y | Y | Y | Y | N | 10 |
| 160 | Wu R et al.2016 | Y | Y | Y | Y | Y | Y | Y | Y | Y | Y | N | 10 |
| 161 | Wu Y et al.2012 | Y | Y | Y | Y | Y | Y | N | Y | Y | Y | N | 9 |
| 162 | Wu Z et al.2019 | Y | Y | Y | Y | Y | Y | Y | Y | Y | Y | N | 10 |
| 163 | Xia S et al.2013 | Y | Y | Y | Y | Y | Y | Y | Y | Y | Y | N | 10 |
| 164 | Xie L et al.2017 | Y | Y | Y | Y | Y | Y | Y | Y | Y | Y | N | 10 |
| 165 | Xu J et al.2018 | Y | Y | Y | Y | Y | Y | Y | Y | Y | Y | N | 10 |
| 166 | Xu L et al.2018 | Y | Y | Y | Y | Y | Y | N | N | Y | Y | N | 8 |
| 167 | Xu S et al.2019 | Y | Y | Y | Y | Y | Y | Y | Y | Y | Y | N | 10 |
| 168 | Xu J et al.2020 | Y | Y | Y | Y | Y | Y | Y | N | Y | Y | N | 9 |
| 169 | Yang H et al.2019 | Y | Y | Y | Y | Y | Y | Y | Y | Y | Y | N | 10 |
| 170 | Yuan L et al.2019 | Y | Y | Y | Y | Y | Y | Y | Y | Y | Y | N | 10 |
| 171 | Zhang H et al.2016 | Y | Y | Y | Y | Y | Y | Y | N | Y | Y | N | 9 |
| 172 | Zhang J et al.2017 | Y | N | Y | Y | Y | Y | Y | Y | Y | Y | N | 9 |
| 173 | Zhang J et al.2019 | Y | Y | Y | Y | Y | Y | Y | Y | Y | Y | N | 10 |
| 174 | Zhang N et al.2018 | Y | Y | Y | Y | Y | Y | Y | Y | Y | Y | N | 10 |
| 175 | Zhang Q et al.2018 | Y | Y | Y | Y | Y | Y | Y | Y | Y | Y | N | 10 |
| 176 | Zhang S et al.2015 | Y | Y | Y | Y | Y | Y | Y | Y | Y | Y | N | 10 |
| 177 | Zhang X et al.2015 | Y | Y | Y | Y | Y | Y | Y | N | Y | Y | N | 9 |
| 178 | Zhang Y et al.2018 | Y | Y | Y | Y | Y | Y | Y | Y | Y | Y | N | 10 |
| 179 | Zhang J et al.2017 | Y | Y | Y | Y | Y | Y | Y | N | Y | Y | N | 9 |
| 180 | Li C et al.2020 | Y | Y | Y | Y | Y | Y | Y | Y | Y | Y | N | 10 |
| 181 | Li J et al.2019 | Y | Y | Y | Y | Y | Y | Y | N | Y | Y | N | 9 |
| 182 | Li W et al.2016 | Y | Y | Y | Y | Y | Y | Y | N | Y | Y | N | 9 |
| 183 | Li X et al.2018 | Y | Y | Y | Y | Y | Y | Y | N | Y | Y | N | 9 |
| 184 | Liu W et al.2014 | Y | Y | Y | Y | Y | Y | Y | Y | Y | Y | N | 10 |
| 185 | Liu X et al.2016 | Y | Y | Y | Y | Y | Y | Y | N | Y | Y | N | 9 |
| 186 | Liu Y et al.2017 | Y | Y | Y | Y | Y | Y | Y | Y | Y | Y | N | 10 |
| 187 | Liu Y et al.2016 | Y | Y | Y | Y | Y | Y | Y | Y | Y | Y | N | 10 |
| 188 | Long X et al.2013 | Y | Y | Y | Y | Y | Y | Y | Y | Y | Y | N | 10 |
| 189 | Luo Y et al.2016 | Y | Y | Y | Y | Y | Y | Y | Y | Y | Y | N | 10 |
| 190 | Ma L et al.2012 | Y | Y | Y | Y | Y | Y | Y | N | Y | Y | N | 9 |
| 191 | Shang J et al.2020 | Y | Y | Y | Y | Y | Y | Y | Y | Y | Y | N | 10 |
| 192 | Shao L et al.2022 | Y | Y | Y | Y | Y | Y | Y | Y | Y | Y | N | 10 |
| 193 | Shi Y et al.2016 | Y | Y | Y | Y | Y | Y | Y | Y | Y | Y | N | 10 |
| 194 | Tan J et al.2012 | Y | Y | Y | Y | Y | Y | Y | Y | Y | Y | N | 10 |
| 195 | Tian C et al.2016 | Y | Y | Y | Y | Y | Y | Y | N | Y | Y | N | 9 |
| 196 | Tong F et al.2018 | Y | Y | Y | Y | Y | Y | Y | N | Y | Y | N | 9 |
| 197 | Li P et al.2023 | Y | Y | Y | Y | Y | Y | Y | Y | Y | Y | N | 10 |
| 198 | Wang T et al.2022 | Y | Y | Y | Y | Y | Y | Y | Y | Y | Y | N | 10 |
| 199 | Hu W et al.2023 | Y | Y | Y | Y | Y | Y | Y | N | Y | Y | N | 9 |
| 200 | Wang C et al.2019 | Y | Y | Y | Y | Y | Y | Y | N | Y | Y | N | 9 |
| 201 | Lang B et al.2023 | Y | Y | Y | Y | Y | Y | Y | N | Y | Y | N | 9 |
| 202 | Yuan H et al.2022 | Y | Y | Y | Y | Y | Y | Y | N | Y | Y | N | 9 |
| 203 | Zheng H et al.2023 | Y | Y | Y | Y | Y | Y | Y | Y | Y | Y | N | 10 |
| 204 | Yang H et al.2023 | Y | Y | Y | Y | Y | Y | Y | N | Y | Y | N | 9 |
| 205 | Yuan H et al.2023 | Y | Y | Y | Y | Y | Y | Y | N | Y | Y | N | 9 |
| 206 | Jiang W et al.2022 | Y | Y | Y | Y | Y | Y | Y | Y | Y | Y | N | 10 |
| 207 | Zhang J et al.2022 | Y | Y | Y | Y | Y | Y | Y | N | Y | Y | N | 9 |
| 208 | Fan F et al.2023 | Y | Y | Y | Y | Y | Y | Y | N | Y | Y | N | 9 |
| 209 | Liang Y et al.2022 | Y | Y | Y | Y | Y | Y | Y | N | Y | Y | N | 9 |
| 210 | Nie L et al.2022 | Y | Y | Y | Y | Y | Y | Y | Y | Y | Y | N | 10 |
| 211 | Qing Z et al.2020 | Y | Y | Y | Y | Y | Y | Y | N | Y | Y | N | 9 |
| 212 | Rao J et al.2022 | Y | Y | Y | Y | Y | Y | Y | N | Y | Y | Y | 10 |
| 213 | Song Y et al.2022 | Y | Y | Y | Y | Y | Y | Y | Y | Y | Y | N | 10 |
| 214 | Sun L et al.2022 | Y | Y | Y | Y | Y | Y | Y | Y | Y | Y | N | 10 |
| 215 | Wang J et al.2023 | Y | Y | Y | Y | Y | Y | Y | N | Y | Y | N | 9 |
| 216 | Lam E et al.2016 | Y | Y | Y | Y | Y | Y | Y | N | Y | Y | N | 9 |
| 217 | Qi Z et al.2013 | Y | Y | Y | Y | Y | Y | Y | N | Y | Y | N | 9 |
| 218 | Zhang F et al.2022 | Y | Y | Y | Y | Y | Y | Y | Y | Y | Y | N | 10 |
| 219 | Zhang X et al.2022 | Y | Y | Y | Y | Y | Y | Y | N | Y | Y | N | 9 |
| 220 | Zhang Y et al.2016 | Y | Y | Y | Y | Y | Y | Y | N | Y | Y | N | 9 |
| 221 | Han Y et al.2022 | Y | Y | Y | Y | Y | Y | Y | N | Y | Y | N | 9 |
| 222 | Ye Z et al.2022 | Y | Y | Y | Y | Y | Y | Y | N | Y | Y | N | 9 |
| 223 | Ma Y et al.2021 | Y | Y | Y | Y | Y | Y | Y | N | Y | Y | N | 9 |
| 224 | Zheng H et al.2014 | Y | Y | Y | Y | Y | Y | Y | N | Y | Y | N | 9 |
| 225 | Zhao X et al.2018 | Y | Y | Y | Y | Y | Y | Y | N | Y | Y | N | 9 |
| 226 | Hu J et al.2022 | Y | Y | Y | Y | Y | Y | Y | Y | Y | Y | Y | 11 |
| 227 | Chang H et al. 2023 | Y | Y | Y | Y | Y | Y | Y | Y | Y | Y | N | 10 |
| 228 | Hou Y et al. 2023 | Y | Y | Y | Y | Y | Y | Y | Y | Y | Y | N | 10 |
| 229 | Lang B et al. 2023 | Y | Y | Y | Y | Y | Y | Y | Y | Y | Y | N | 10 |
| 230 | Liu J et al. 2023 | Y | Y | Y | Y | Y | Y | Y | Y | Y | Y | N | 10 |
| 231 | Yi J et al. 2023 | Y | Y | Y | Y | Y | Y | Y | N | Y | Y | N | 9 |
| 232 | Zhang J et al. 2023 | Y | Y | Y | Y | Y | Y | Y | Y | Y | Y | N | 10 |
| 233 | Zhou X et al. 2023 | Y | Y | Y | Y | Y | Y | Y | Y | Y | Y | N | 10 |
| 234 | Zhu Y et al. 2023 | Y | N | N | Y | Y | Y | Y | Y | Y | Y | N | 8 |
| 235 | Cui Y et al. 2023 | Y | Y | Y | Y | Y | Y | Y | Y | Y | Y | N | 10 |
| 236 | Fan J et al. 2018 | Y | Y | Y | Y | Y | Y | Y | N | Y | Y | N | 9 |
| 237 | Fang X et al. 2015 | Y | Y | Y | Y | Y | Y | Y | Y | Y | Y | N | 10 |
| 238 | Gao L et al. 2020 | Y | Y | Y | Y | Y | Y | Y | N | Y | Y | N | 9 |
| 239 | Han T et al. 2013 | Y | Y | Y | Y | Y | Y | Y | N | Y | Y | Y | 10 |
| 240 | Han T et al. 2015 | Y | Y | Y | Y | Y | Y | Y | Y | Y | Y | N | 10 |
| 241 | Han Z et al. 2016 | Y | Y | Y | Y | Y | Y | Y | Y | Y | Y | N | 10 |
| 242 | Huang L et al. 2018 | Y | Y | Y | Y | Y | Y | Y | N | Y | Y | N | 9 |
| 243 | Li F et al. 2023 | Y | Y | Y | Y | Y | Y | Y | Y | Y | Y | N | 10 |
| 244 | Li L et al. 2017 | Y | Y | Y | Y | Y | Y | Y | Y | Y | Y | N | 10 |
| 245 | Li L et al. 2023 | Y | Y | Y | Y | Y | Y | Y | Y | Y | Y | N | 10 |
| 246 | Li Y et al. 2023 | Y | Y | Y | Y | Y | Y | Y | Y | Y | Y | N | 10 |
| 247 | Lin R et al. 2015 | Y | Y | Y | Y | Y | Y | Y | N | N | Y | N | 8 |
| 248 | Liu Y et al. 2023 | Y | Y | Y | Y | Y | Y | Y | Y | Y | Y | N | 10 |
| 249 | Liu Z et al. 2018 | Y | Y | Y | Y | Y | Y | Y | N | Y | Y | N | 9 |
| 250 | Long Y et al. 2014 | Y | Y | Y | Y | Y | Y | Y | N | Y | Y | N | 9 |
| 251 | Abudukelimu N et al. 2023 | Y | Y | Y | Y | Y | Y | Y | Y | Y | Y | N | 10 |
| 252 | Shi Z et al. 2019 | Y | Y | Y | Y | Y | Y | Y | N | Y | Y | N | 9 |
| 253 | Sun X et al. 2023 | Y | Y | Y | Y | Y | Y | Y | Y | Y | Y | N | 10 |
| 254 | Tang X 2017 | Y | Y | Y | Y | Y | Y | Y | N | Y | Y | N | 9 |
| 255 | Wang D et al. 2023 | Y | Y | Y | Y | Y | Y | Y | Y | Y | Y | N | 10 |
| 256 | Wang H 2016 | Y | Y | Y | Y | Y | Y | Y | Y | Y | Y | N | 10 |
| 257 | Wang J et al. 2018 | Y | Y | Y | Y | Y | Y | Y | N | Y | Y | N | 9 |
| 258 | Wang J et al. 2020 | Y | Y | Y | Y | Y | Y | Y | Y | Y | Y | N | 10 |
| 259 | Wang M et al. 2023 | Y | Y | Y | Y | Y | Y | Y | Y | Y | Y | N | 10 |
| 260 | Wang R 2020 | Y | Y | Y | Y | Y | Y | Y | Y | Y | Y | N | 10 |
| 261 | Wang T 2022 | Y | Y | Y | Y | Y | Y | Y | N | Y | Y | Y | 10 |
| 262 | Wei F et al. 2018 | Y | Y | Y | Y | Y | Y | Y | N | Y | Y | N | 9 |
| 263 | Wu M et al. 2020 | Y | Y | Y | Y | Y | Y | Y | Y | Y | Y | N | 10 |
| 264 | Xie Q et al. 2022 | Y | Y | Y | Y | Y | Y | Y | N | Y | Y | N | 9 |
| 265 | Xu Z et al. 2013 | Y | Y | Y | Y | Y | Y | Y | Y | Y | Y | N | 10 |
| 266 | Yang S et al. 2018 | Y | Y | Y | Y | Y | Y | Y | N | Y | Y | N | 9 |
| 267 | Yang X et al. 2015 | Y | Y | Y | Y | Y | Y | Y | N | Y | Y | N | 9 |
| 268 | Yang Y et al. 2016 | Y | Y | Y | Y | Y | Y | Y | N | Y | Y | N | 9 |
| 269 | Zhang D 2014 | Y | Y | Y | Y | Y | Y | Y | Y | Y | Y | N | 10 |
| 270 | Zhang J et al. 2017 | Y | Y | Y | Y | Y | Y | Y | Y | Y | Y | N | 10 |
| 271 | Zhang J et al. 2015 | Y | Y | Y | Y | Y | Y | Y | Y | Y | Y | N | 10 |
| 272 | Zhang L et al. 2020 | Y | Y | Y | Y | Y | Y | Y | Y | Y | Y | N | 10 |
| 273 | Zhang T et al. 2023 | Y | Y | Y | Y | Y | Y | Y | Y | Y | Y | N | 10 |
| 274 | Zou C 2016 | Y | Y | Y | Y | Y | Y | Y | N | Y | Y | N | 9 |
| 275 | Zheng W et al. 2024 | Y | Y | Y | Y | Y | Y | Y | N | Y | Y | N | 9 |
| 276 | Tian L et al. 2024 | Y | Y | Y | Y | Y | Y | Y | N | Y | Y | N | 9 |
| 277 | Shen P et al. 20224 | Y | Y | Y | Y | Y | Y | Y | N | Y | Y | N | 9 |
| 278 | Na Z et al. 2024 | Y | Y | Y | Y | Y | Y | Y | N | Y | Y | N | 9 |
| 279 | Meng Y et al. 2024 | Y | Y | Y | Y | Y | Y | Y | Y | Y | Y | N | 10 |
| 280 | Lv Z et al. 2024 | Y | Y | Y | Y | Y | Y | Y | N | Y | Y | N | 9 |
| 281 | Lv Y et al. 2024 | Y | Y | Y | Y | Y | Y | Y | N | Y | Y | N | 9 |
| 282 | Lan Q et al. 2024 | Y | Y | Y | Y | Y | Y | Y | N | Y | Y | N | 9 |
| 283 | Hu J et al. 2024 | Y | Y | Y | Y | Y | Y | Y | N | Y | Y | N | 9 |
| 284 | He P et al. 2024 | Y | Y | Y | Y | Y | Y | Y | N | Y | Y | N | 9 |
| 285 | Chen B et al. 2024 | Y | Y | Y | Y | Y | Y | Y | N | Y | Y | N | 9 |
| 286 | Zheng L et al. 2024 | Y | Y | Y | Y | Y | Y | Y | N | Y | Y | N | 9 |
| 287 | Zhang J et al. 2024 | Y | Y | Y | Y | Y | Y | Y | Y | Y | Y | Y | 11 |
| 288 | Zhang J et al. 2024 | Y | Y | Y | Y | Y | Y | Y | Y | Y | Y | N | 10 |
| 289 | Yi X et al. 2024 | Y | Y | Y | Y | Y | Y | Y | N | Y | Y | N | 9 |
| 290 | Wang S et al. 2024 | Y | Y | Y | Y | Y | Y | Y | N | Y | Y | N | 9 |
| 291 | Wang J et al. 2024 | Y | Y | Y | Y | Y | Y | Y | N | Y | Y | N | 9 |
| 292 | Tian J et al. 2024 | Y | Y | Y | Y | Y | Y | Y | N | Y | Y | N | 9 |
| 293 | Liu P et al. 2024 | Y | Y | Y | Y | Y | Y | Y | Y | Y | Y | N | 10 |
| 294 | Huang Z et al. 2024 | Y | Y | Y | Y | Y | Y | Y | N | Y | Y | N | 9 |
| 295 | Huang Y et al. 2024 | Y | Y | Y | Y | Y | Y | Y | Y | Y | Y | N | 10 |
| 296 | Fan X et al. 2024 | Y | Y | Y | Y | Y | Y | Y | N | Y | Y | N | 9 |

*Q1. Define the source of information (survey, record review); Q2. List inclusion and exclusion criteria for exposed and unexposed subjects (cases and controls) or refer to previous publications; Q3. Indicate time period used for identifying patients; Q4. Indicate whether or not subjects were consecutive if not population-based; Q5. Indicate if evaluators of subjective components of study were masked to other aspects of the status of the participants; Q6. Describe any assessments undertaken for quality assurance purposes (e.g., test/retest of primary outcome measurements); Q7. Explain any patient exclusions from analysis; Q8. Describe how confounding was assessed and/or controlled; Q9. If applicable, explain how missing data were handled in the analysis; Q10. Summarize patient response rates and completeness of data collection; Q11. Clarify what follow-up, if any, was expected and the percentage of patients for which incomplete data or follow-up was obtained. Y indicates that this item was yes in this study; N indicates that this item was no in this study; and U indicates that this item was no in this study.

***Appendix4: Meta-regression results***

Univariate Meta regression results

| Univariate Meta regression | Outpatients | | | | Health checkups | | | |
| --- | --- | --- | --- | --- | --- | --- | --- | --- |
|  | exp(b) | Std.Err. | Coef. | p-value | exp(b) | Std.Err. | Coef. | p-value |
| Region | 0.9759791 | 0.0297203 | -0.0243141 | 0.425 | 1.597189 | 0.2727693 | 0.4682453 | 0.012 |
| Sampling type | 0.803325 | 0.0432562 | -0.2189959 | <0.001 | 1.209207 | 0.1608099 | 0.1899649 | 0.167 |
| Sampling site | 1.086071 | 0.0273498 | 0.082567 | 0.001 | 1.331469 | 0.1179426 | 0.2862828 | 0.004 |
| MSM | 0.7228233 | 0.0721087 | -0.3245906 | 0.001 | / | / | / | / |
| Diagnosed with cancer or not | 2.093566 | 0.3052126 | 0.7388688 | <0.001 | / | / | / | / |
| Publication year | 0.9821146 | 0.0120621 | 0.0180473 | 0.143 | 0.9705915 | 0.0543443 | -0.0298496 | 0.599 |
| Sample size(groups) | 0.7085554 | 0.0624164 | -0.344527 | <0.001 | 0.6522702 | 0.2395971 | -0.4272965 | 0.257 |

***Appendix5: Characteristics of studies examining the prevalence of HPV positivity among Chinese males***

|  | Author, year | Region | Sampling time | design | Study setting | Study population | HPV DNA detection method | Primer type (name or location) | Anatomical site | Samples tested for HPV | Age (years) | Age range, years | | Any HPV genotype n (%) | | High-risk genotype n (%) | | Low-risk genotype n (%) | | | Overall analysis | | Type analysis | | Age analysis |
| --- | --- | --- | --- | --- | --- | --- | --- | --- | --- | --- | --- | --- | --- | --- | --- | --- | --- | --- | --- | --- | --- | --- | --- | --- | --- |
| 1 | Cao F et al.2014 | Eastern | Dec. 2006 - Jan. 2008 | Cross-sectional | Hospital | ESCC | in situ hybridization | - | Head and neck | 84 |  |  | 24 (28.57%) | | | - | | - | | | Y | | Y | | N |
| 2 | Chen L et al.2021 | Eastern | June 2016 - July 2019 | Cross-sectional | Hospital | SCCLHP | ddPCR | primer-prob | Head and neck | 110 |  |  | 18 (16.36%) | | | 15 (13.64%) | | 5 (4.55%) | | | Y | | Y | | N |
| 3 | Chen X et al.2016 | Eastern | Jan. 2014 - May 2015 | Cross-sectional | Clinic(male with genital warts) | anogenital warts | Unknown | PGMY09/11 | Anal | 935 | Median42 |  | 447 (47.81%) | | | 230 (24.60%) | | 356 (38.07%) | | | Y | | Y | | Y |
| 4 | Cheng S et al.2012 | Taiwan | Mar. 2010 - Dec. 2010 | Case-control | Clinic(MSM with HIV) | MSM | PCR-reverse blot hybridization | biotinylated | Anal | 405 | Median31 |  | 156 (38.52%) | | | 108 (26.67%) | | 114 (28.15%) | | | Y | | N | | N |
| 5 | Cheng S et al.2018 | Taiwan | Mar. 2011 - June 2016 | Cross-sectional | Clinic(male with HIV)) | HIV Positive | PCR-reverse blot hybridization | biotinylated | Genital | 714 | Mean30.7(SD8.2) |  | 610 (85.43%) | | | 509 (71.29%) | | 529 (74.09%) | | | Y | | N | | N |
| 6 | Cheng Y et al.2012 | Taiwan | May 2003 - 2006 | Cross-sectional | Clinic(male with genital warts) | condyloma acuminata (CA) | Gene chip | MY11/GP6t | mutiple sites | 100 | Mean35.8 | 18-82 | 100 (100.00%) | | | - | | - | | | Y | | Y | | N |
| 7 | Chu C et al.2020 | Eastern | Jan. 1999 - Dec. 2013 | Cross-sectional | Hospital | PC | FQ-PCR | - | Genital | 226 | Median52 | 24-86 | 74 (32.74%) | | | 71 (31.42%) | | 3 (1.33%) | | | Y | | Y | | N |
| 8 | Cong X et al.2016 | Northeastern | Nov. 2001 - Oct. 2003 | Cross-sectional | Hospital | CA | Unknown | MY09/MY11 | Genital | 50 | Median37 | 20-84 | 49 (98.00%) | | | - | | - | | | Y | | Y | | N |
| 9 | Cui L et al.2019 | Eastern | Jan. 2005 - Dec. 2011 | Cross-sectional | Hospital | LSCC | Reverse Dot Blot | - | Head and neck | 307 |  |  | 38 (12.38%) | | | - | | - | | | Y | | N | | N |
| 10 | Fan S et al.2020 | Multiple | Jan. 2019 - Apr. 2019 | Cross-sectional | Clinic and Community(MSM) | MSM | Unknown | - | Genital | 426 | Median20 | 17-27 | 160 (37.56%) | | | 107 (25.12%) | | 112 (26.29%) | | | Y | | Y | | Y |
| 11 | Gu W et al.2020 | Eastern | Jan. 2006 - Dec. 2017 | Cross-sectional | Hospital | PC | Reverse Dot Blot | - | Genital | 340 | Median56 |  | 166 (48.82%) | | | 163 (47.94%) | | - | | | Y | | Y | | Y |
| 12 | He ZH et al.2013 | Central | Jan. 2007 - Dec. 2009 | Cross-sectional | Rural community | HNSCC | Reverse Dot Blot | SPF1/GP6+ | Anal | 2236 | Median42 |  | 391 (17.49%) | | | 140 (6.26%) | | 251 (11.23%) | | | Y | | Y | | N |
| 13 | Hu JM et al.2013 | Western | Jan. 2000 - Dec. 2006 | Case-control | Hospital | ESCC | Reverse Dot Blot | nested primer | Head and neck | 93 |  |  | - | | | - | | - | | | N | | Y | | N |
| 14 | Hu Y et al.2013 | Eastern | Jan. 2010 - July 2011 | Cross-sectional | Clinic and Community(MSM) | MSM | Suspension bead array method | - | Genital | 671 | Mean28 | 18-61 | 438 (65.28%) | | | 312 (46.50%) | | 126 (18.78%) | | | Y | | Y | | N |
| 15 | Lee L et al.2015 | Taiwan | Jan. 2004 - Dec. 2011 | Cohort | Hospital | OSCC | MeltPro HPV Genotyping Assay | - | Head and neck | 938 |  |  | 182 (19.40%) | | | - | | - | | | Y | | N | | N |
| 16 | Li P et al.2021 | Multiple | Sep. 2018 - May 2019 | Cross-sectional | CDC and Community(MSM) | MSM | Unknown | - | Genital | 265 | Median20 | 17-24 | 96 (36.23%) | | | 63 (23.77%) | | 65 (24.53%) | | | Y | | Y | | Y |
| 17 | Li S et al.2017 | Eastern | Mar. 2013 - Dec. 2015 | Cross-sectional | Hospital | ESCC | Reverse Dot Blot | - | Head and neck | 136 |  |  | 55 (40.44%) | | | - | | - | | | Y | | N | | N |
| 18 | Li W et al.2017 | Eastern | Jan. 2010 - Dec. 2015 | Cross-sectional | Clinic and Hospital | outpatients | Flow-FISH | - | Anal | 1432 | Mean34(SD10.5) |  | 367 (25.63%) | | | - | | - | | | Y | | Y | | N |
| 19 | Li X et al.2016 | Mutiple sites | Mar. 2012 - Dec. 2013 | Cross-sectional | STD clinic and Community(MSM with HIV-infected and uninfected) | MSM | Flow-through Hybridization | - | Genital | 822 | Mean32.2 |  | 537 (65.33%) | | | 419 (50.97%) | | - | | | Y | | Y | | N |
| 20 | Li X et al.2021 | Eastern | Jan. 2015 - Dec. 2019 | Cross-sectional | Clinic | outpatients | Flow-FISH | - | mutiple sites | 1227 |  |  | 804 (65.53%) | | | 344 (28.04%) | | 707 (57.62%) | | | Y | | Y | | N |
| 21 | Li Y et al.2021 | Eastern | Dec. 2017 - Sep. 2020 | Cross-sectional | Hospital | outpatients | FQ-PCR | - | mutiple sites | 333 |  |  | 78 (23.42%) | | | - | | - | | | Y | | Y | | Y |
| 22 | Li Z et al.2015 | Western | Apr. 2014 - July 2014 | Cross-sectional | Hospital | HIV Positive MSM | Flow-FISH | - | Genital | 193 | Mean34(SD9) | 18-60 | 191 (98.96%) | | | 183 (94.82%) | | 154 (79.79%) | | | Y | | Y | | Y |
| 23 | Lin C et al.2018 | Taiwan | Jan. 2013 - Dec. 2016 | Cross-sectional | Hospital | MSM | PCR-reverse blot hybridization | - | mutiple sites | 279 | Median26(SD5.9) |  | 115 (41.22%) | | | - | | - | | | Y | | Y | | N |
| 24 | Liu T et al.2013 | Western | Jan. 2005 - Dec. 2008 | Cross-sectional | hospital | SSCC | FQ-PCR | GP5+/GP6+ | Head and neck | 153 |  |  | - | | | - | | - | | | N | | Y | | N |
| 25 | Liu X et al.2019 | Eastern | Aug. 2016 - Oct. 2017 | Cohort | CDC (male with HIV) | outpatients | Flow-through Hybridization | - | Genital | 273 | Mean42.3(SD12.7) |  | 106 (38.83%) | | | 92 (33.70%) | | - | | | Y | | Y | | N |
| 26 | Luo Z et al.2015 | Eastern | Aug. 2009 - Oct. 2014 | Cross-sectional | Men attending Chaozhou Central Hospital. | anogenital warts | Geno Array HPV | - | mutiple sites | 305 | Mean33.3(10.2) | 16-65 | - | | | 120 (39.34%) | | 282 (92.46%) | | | N | | Y | | N |
| 27 | Ma D et al.2019 | Eastern | Jan. 2016 - Dec. 2018 | Cross-sectional | STI clinic | outpatients | FQ-PCR | - | Anal | 1163 | Median35(12.3) | 18-67 | 489 (42.05%) | | | 319 (27.43%) | | - | | | Y | | Y | | Y |
| 28 | Meng H et al.2018 | Northeastern | Jan. 2000 - Feb. 2016 | Cross-sectional | Hospital | OPSCC | Unknown | - | Head and neck | 1167 |  |  | 69 (5.91%) | | | - | | - | | | Y | | N | | N |
| 29 | Mujtaba H et al.2018 | Western | Jan. 2013 - Feb. 2018 | Cross-sectional | Hospital | tonsillectomy | Gene chip | Gp5+/GP6+ | Head and neck | 226 |  |  | 6 (2.65%) | | | 3 (1.33%) | | 3 (1.33%) | | | Y | | Y | | N |
| 30 | Ni Y et al.2022 | Eastern | Apr. 2020 - Oct. 2020 | Cross-sectional | Clinic and Community(MSM) | MSM | MassARRAY (Agena Bioscience) technique | GP5+/GP6+ | mutiple sites | 211 | Mean31(7.9) |  | 103 (48.82%) | | | 79 (37.44%) | | 40 (18.96%) | | | Y | | N | | N |
| 31 | Ning H et al.2015 | Eastern | Dec. 2012 - Nov. 2013 | Cross-sectional | Hospital | CA | Flow-through Hybridization | - | mutiple sites | 110 |  | 16-65 | 107 (97.27%) | | | 48 (43.64%) | | 100 (90.91%) | | | Y | | Y | | N |
| 32 | Peng J et al.2013 | Eastern | Mar. 2010 - July 2010 | Cross-sectional | Hospital | MSM | matrix-assisted laser desorption ionization-time of flight mass spectrometry (MALDI-TOF MS) method | MY09/MY11 | Genital | 200 |  |  | 129 (64.50%) | | | - | | - | | | Y | | N | | N |
| 33 | Peng J et al.2016 | Eastern | - | Cross-sectional | Clinic and Community(MSM) | MSM | MassARRAY MALDI-TOF MS | - | Genital | 224 | Mean29.5(SD8.9) | 18-61 | 204 (91.07%) | | | - | | - | | | Y | | N | | N |
| 34 | Qian, H et al.2017 | Eastern | - | Cross-sectional | Clinic and Community(MSM) | MSM | Suspension bead array method | - | Genital | 671 | Median29 | 25-34 | 438 (65.28%) | | | - | | - | | | Y | | N | | N |
| 35 | Ren X et al.2017 | Eastern | Jan. 2013 - Oct. 2013 | Cross-sectional | STD clinic and Community(MSM with HIV) | MSM | Reverse Dot Blot | MY09/11 | Genital | 164 | Median26 |  | 105 (64.02%) | | | 63 (38.41%) | | 71 (43.29%) | | | Y | | Y | | N |
| 36 | Strong C et al.2020 | Taiwan | Oct. 2015 - May 2016 | Cohort | Community and social media | MSM | PCR-reverse blot hybridization | - | mutiple sites | 253 | 64% 20-29 |  | 87 (34.39%) | | | 62 (24.51%) | | 47 (18.58%) | | | Y | | Y | | Y |
| 37 | Su Y et al.2019 | Western | May 2014 - Mar. 2016 | Cross-sectional | Community and college | health checkups | MeltPro HPV Genotyping Assay | - | mutiple sites | 383 | Median42 |  | 37 (9.66%) | | | 37 (9.66%) | | 0 (0.00%) | | | Y | | Y | | N |
| 38 | Tong F et al.2018 | Northeastern | Jan. - Dec. 2016 | Cohort | Hospital | LSCC | in situ hybridization | - | Head and neck | 181 |  |  | 113 (62.43%) | | | - | | - | | | Y | | N | | N |
| 39 | Wang C et al.2019 | Taiwan | Jan. 2016 - Dec. 2016 | Cross-sectional | Hospital | Oral lesions | Gene chip | MY11/biotinylated GP6t (192 bp) | Head and neck | 92 |  |  | 3 (3.26%) | | | 2 (2.17%) | | 2 (2.17%) | | | Y | | Y | | Y |
| 40 | Wang H et al.2021 | Central | June 2015 - June 2020 | Cross-sectional | Clinic | outpatients | Flow-through Hybridization | - | Anal | 3690 | Mean41.6(SD11.3) | 20-85 | 1106 (29.97%) | | | - | | - | | | Y | | Y | | Y |
| 41 | Wang Y et al.2013 | Central | Dec. 2007 - Dec. 2008 | Cross-sectional | Hospital | ESCC | in situ hybridization | - | Head and neck | 56 |  |  | 9 (16.07%) | | | - | | - | | | Y | | N | | N |
| 42 | Wei F et al.2016 | Western | Mar. 2014 - July 2014 | Cross-sectional | Rural community and urban community | outpatients | FQ-PCR | GP5+/6+ | mutiple sites | 1937 | Median38 |  | 204 (10.53%) | | | 183 (9.45%) | | - | | | Y | | Y | | N |
| 43 | Wong M et al.2018 | Hong kong and Macau | - | Cross-sectional | Community | outpatients | Reverse Dot Blot | - | Head and neck | 680 |  |  | 149 (21.91%) | | | - | | - | | | Y | | N | | N |
| 44 | Wu P et al.2020 | Taiwan | Mar. 2015 - July 2016 | Cross-sectional | Clinic | MSM | Unknown | - | Genital | 139 | Median31 |  | 92 (66.19%) | | | 74 (53.24%) | | 66 (47.48%) | | | Y | | Y | | N |
| 45 | Xiang J et al.2021 | Eastern | Jan. 2017 - Sep. 2019 | Cross-sectional | STD clinic | outpatients | Reverse Dot Blot | - | Anal | 1711 | Mean34.7(SD9.5) | 17-89 | 869 (50.79%) | | | - | | - | | | Y | | Y | | N |
| 46 | Xin H et al.2017 | Eastern | July 2015 - Oct. 2016 | Cross-sectional | STD clinic | outpatients | Flow-through Hybridization+Gene chip | - | Anal | 198 | Mean33(SD12) | 18-66 | 88 (44.44%) | | | - | | - | | | Y | | Y | | Y |
| 47 | Xu T et al.2020 | Eastern | Jan. 2007 - July 2019 | Cross-sectional | Hospital | OPSCC | in situ hybridization | - | Head and neck | 127 |  |  | 78 (61.42%) | | | - | | - | | | Y | | N | | N |
| 48 | Xu Y et al.2014 | Eastern | Apr. 2006 - | Cohort | Hospital | LSCC | Geno Array HPV | - | Head and neck | 654 |  |  | 33 (5.05%) | | | 33 (5.05%) | | - | | | Y | | Y | | N |
| 49 | Xu Y et al.2015 | Eastern | Apr. 2004 - Apr. 2013 | Cross-sectional | Hospital | HNSCC | Geno Array HPV | L1 | Head and neck | 229 |  |  | 8 (3.49%) | | | 8 (3.49%) | | - | | | Y | | N | | N |
| 50 | Xu Y et al.2021 | Western | Jan. 2015 - May 2020 | Cross-sectional | Clinic | outpatients | Flow-through Hybridization | - | Anal | 369 | Mean36.2(SD9.1) |  | 88 (23.85%) | | | - | | - | | | Y | | Y | | Y |
| 51 | Yan Y et al.2021 | Eastern | Jan. 2015 - Dec. 2019 | Cross-sectional | Hospital | Bladder Cancer | Capture Hybridization | - | Others | 122 | Mean66.4 | 32-89 | 33 (27.05%) | | | 33 (27.05%) | | - | | | Y | | Y | | N |
| 52 | Yang Y et al.2013 | Eastern | - | Cross-sectional | Hospital | infertile male&health checkups | Suspension bead array method | - | Sperm | 1138(615+523) | Mean31.2 | 21-48 | 142 (12.48%) | | | - | | - | | | Y | | Y | | N |
| 53 | Yang Y et al.2012 | Eastern | Jan. 2011 - Apr. 2011 | Cross-sectional | Community and social media(MSM with HIV) | HIV Positive MSM | Suspension bead array method | - | Genital | 95 | 3.2%≤19 |  | 36 (37.89%) | | | - | | - | | | Y | | Y | | Y |
| 54 | Ye Z et al.2021 | Northeastern | July 2020 - Dec. 2020 | Cross-sectional | Clinic(MSM) | MSM | FQ-PCR | - | Genital | 203 | Median30 |  | 121 (59.61%) | | | 99 (48.77%) | | - | | | Y | | N | | N |
| 55 | Yin W et al.2020 | Eastern | Jan. 2014 - Sep. 2018 | Trial | Hospital | outpatients | Flow-through Hybridization | - | Anal | 1044 |  | 15-83 | 567 (54.31%) | | | 347 (33.24%) | | 385 (36.88%) | | | Y | | Y | | Y |
| 56 | Yu C et al.2013 | Taiwan | Aug. 2010 - Feb. 2011 | Cross-sectional | Clinic(male with HIV) | HIV positive population | Unknown | MP09/MP11/GP6 | Genital | 194 | Mean38.4(SD11.9) | 20-68 | 143 (73.71%) | | | 86 (44.33%) | | 66 (34.02%) | | | Y | | Y | | N |
| 57 | Yu Y et al.2013 | Western | Jan. 2005 - Dec. 2008 | Cross-sectional | Hospital | Lung SCC | Unknown | - | Others | 108 | Mean55.9(SD12.3) |  | 46 (42.59%) | | | - | | - | | | Y | | N | | N |
| 58 | Zhang Y et al.2017 | Eastern | Jan. 2005 - Dec. 2014 | Cross-sectional | Hospital | Inverting papilloma | Geno Array HPV | - | Head and neck | 73 |  |  | 48 (65.75%) | | | 52 (71.23%) | | 47 (64.38%) | | | Y | | Y | | N |
| 59 | Zhang C et al.2018 | Eastern | Oct. 2015 - Mar. 2017 | Cross-sectional | Clinic | outpatients | Gene chip | - | Anal | 1205 | Median33 | 16-67 | 704 (58.42%) | | | 375 (31.12%) | | 412 (34.19%) | | | Y | | Y | | Y |
| 60 | Zhang C et al.2017 | Central | Jan. 2012 - Dec. 2015 | Cohort | Population | General population | FQ-PCR | - | Head and neck | 1361 |  |  | 1266 (93.02%) | | | - | | - | | | Y | | N | | N |
| 61 | Zhang C et al.2015 | Central | Jan. 2007 - Dec. 2009 | Cohort | Population | General population | FQ-PCR | - | mutiple sites | 2681 | Median42 |  | 1772 (66.09%) | | | 832 (31.03%) | | - | | | Y | | N | | N |
| 62 | Zhang D et al.2014 | Eastern | July 2009 - Sep. 2009 | Cross-sectional | STD clinic（MSM） | MSM | Gene chip | - | Genital | 408 |  |  | 149 (36.52%) | | | - | | - | | | Y | | Y | | N |
| 63 | Zhang J et al.2022 | Eastern | Jan. 2016 - Dec. 2019 | Cross-sectional | CDC(male with HIV) | HIV Positive MSM | Flow-through Hybridization | - | Genital | 766 | Mean46.9 | 18-82 | 373 (48.69%) | | | 309 (40.34%) | | 173 (22.58%) | | | Y | | Y | | N |
| 64 | Zhang X et al.2013 | Eastern | May 2010 - July 2010 | Cross-sectional | Community and social media | MSM | Flow-FISH | - | Genital | 287 |  |  | 205 (71.43%) | | | - | | - | | | Y | | N | | Y |
| 65 | Zhong Y et al.2019 | Eastern | Aug. 2016 - July 2018 | Cross-sectional | Hospital | health checkups | FQ-PCR | - | Anal | 621 | Mean38.3(11.6) | 18-55 | 18 (2.90%) | | | 17 (2.74%) | | 3 (0.48%) | | | Y | | Y | | Y |
| 66 | Zhou Y et al.2022 | Eastern | Jan. 2017 - | Cohort | Hospital | MSM | Unknown | - | Genital | 196 | Mean27.3 |  | 77 (39.29%) | | | 42 (21.43%) | | 35 (17.86%) | | | Y | | Y | | N |
| 67 | Zhou Y et al.2020 | Eastern | Jan. 2017 - Aug. 2017 | Cross-sectional | Clinic | MSM | Flow-through Hybridization | - | Genital | 536 | Mean26.4 |  | 263 (49.07%) | | | 207 (38.62%) | | 145 (27.05%) | | | Y | | Y | | N |
| 68 | Chang Y et al.2021 | Eastern | Apr. 2009 - Oct. 2020 | Cross-sectional | Clinic | Spouses of HPV-infected females | Unknown | - | Anal | 643 | Mean47 | 15-65 | 199 (30.95%) | | | - | | - | | | Y | | Y | | N |
| 69 | Chen T et al.2018 | Eastern | Dec. 2013 - June 2016 | Cross-sectional | Clinic | HIV Positive MSM | Reverse Dot Blot | - | Genital | 125 | Mean32.5 | 18-45 | 87 (69.60%) | | | - | | - | | | Y | | Y | | N |
| 70 | Chen W et al.2016 | Western | Aug. 2012 - May 2015 | Cross-sectional | Clinic | Suspected CA | Flow-through Hybridization | - | Anal | 218 |  |  | 134 (61.47%) | | | 58 (26.61%) | | 109 (50.00%) | | | Y | | Y | | Y |
| 71 | Chen Y et al.2014 | Central | June 2011 - Mar. 2012 | Cross-sectional | Clinic | outpatients | Flow-through Hybridization | - | Anal | 525 |  |  | 192 (36.57%) | | | 68 (12.95%) | | 124 (23.62%) | | | Y | | N | | N |
| 72 | Chen Z et al.2016 | Western | June 2014 - June 2015 | Cross-sectional | Hospital | CA | Unknown | MY09/MY11 | mutiple sites | 94 |  |  | 85 (90.43%) | | | - | | - | | | Y | | N | | Y |
| 73 | Deng D et al.2019 | Western | May 2011 - Dec. 2018 | Cross-sectional | Hospital | outpatients | Gene chip | - | Anal | 250 |  |  | 122 (48.80%) | | | 63 (25.20%) | | 83 (33.20%) | | | Y | | Y | | N |
| 74 | Deng G et al.2016 | Eastern | - | Cross-sectional | Clinic | CA | Unknown | - | Anal | 25 |  |  | 25 (100.00%) | | | 12 (48.00%) | | 20 (80.00%) | | | Y | | N | | N |
| 75 | Deng J et al.2014 | Western | May 2009 - Jan. 2013 | Cross-sectional | Clinic | outpatients | Gene chip | - | Anal | 259 | Mean35.6(13.3) | 15-82 | 202 (77.99%) | | | 71 (27.41%) | | 192 (74.13%) | | | Y | | N | | Y |
| 76 | Dong z et al.2016 | Eastern | Jan. 2014 - Dec. 2015 | Cross-sectional | Clinic | outpatients | Flow-through Hybridization | - | Anal | 500 |  | 18-65 | 217 (43.40%) | | | 80 (16.00%) | | 183 (36.60%) | | | Y | | N | | Y |
| 77 | Fang B et al.2018 | Eastern | Jan. 2016 - Dec. 2017 | Cross-sectional | Hospital | CA | Flow-through Hybridization | - | Anal | 551 | Mean31.7(SD10.4) |  | 252 (45.74%) | | | - | | - | | | Y | | Y | | Y |
| 78 | Feng L et al.2019 | Eastern | Jan. 2014 - Dec. 2016 | Cross-sectional | Hospital | CA | Reverse Dot Blot | - | Anal | 4500 |  | 17-74 | 2799 (62.20%) | | | 1755 (39.00%) | | 2283 (50.73%) | | | Y | | Y | | Y |
| 79 | Gao M et al.2019 | Eastern | Dec. 2017 - Aug. 2018 | Cross-sectional | Hospital（MSM with HIV） | HIV Positive MSM | Gene chip | - | Genital | 140 |  | 18-60 | 109 (77.86%) | | | 81 (57.86%) | | 86 (61.43%) | | | Y | | Y | | Y |
| 80 | Guo S et al.2018 | Central | Jan. 2014 - Oct. 2016 | Cross-sectional | Hospital | papilloma of external auditory canal | in situ hybridization | - | Head and neck | 34 |  |  | 34 (100.00%) | | | 7 (20.59%) | | 27 (79.41%) | | | Y | | N | | N |
| 81 | Hao G et al.2022 | Western | July 2017 - July 2020 | Cross-sectional | Clinic | outpatients | FQ-PCR | - | Anal | 618 | Mean36.5(SD5) | 13-68 | 256 (41.42%) | | | 171 (27.67%) | | 146 (23.62%) | | | Y | | Y | | Y |
| 82 | Huang J et al.2015 | Eastern | Aug. 2011 - Oct. 2012 | Cross-sectional | Clinic | outpatients | Flow-through Hybridization | - | Anal | 382 | Mean32(SD11.7) | 18-65 | 142 (37.17%) | | | 47 (12.30%) | | - | | | Y | | Y | | Y |
| 83 | Huang Y et al.2015 | Eastern | Aug. 2012 - Mar. 2014 | Cross-sectional | Clinic | outpatients | FQ-PCR | - | Anal | 200 | Mean35.1(SD17.3) | 15-84 | 66 (33.00%) | | | - | | - | | | Y | | Y | | N |
| 84 | Huang Z et al.2013 | Eastern | May 2011 - Oct. 2013 | Cross-sectional | Clinic | CA | Flow-through Hybridization | - | Anal | 223 | Median32 | 16-78 | 190 (85.20%) | | | - | | - | | | Y | | Y | | Y |
| 85 | Xi M et al.2022 | Western | Sep. 2016 - Dec. 2020 | Cross-sectional | Hospital | MSM | Unknown | - | Genital | 918 | Mean32.9(SD8) |  | 451 (49.13%) | | | 322 (35.08%) | | 241 (26.25%) | | | Y | | N | | N |
| 86 | Zhao M et al.2013 | Central | June 2011 - June 2012 | Cross-sectional | CDC(MSM with HIV) | HIV positive MSM&CA | Reverse Dot Blot | - | mutiple sites | 257 (67+157) | Mean32.4(SD14.2) | 20-71 | 224 (87.16%) | | | 54 (21.01%) | | 170 (66.15%) | | | Y | | N | | N |
| 87 | Fu Y et al.2018 | Northeastern | Jan. 2016 - Jan. 2017 | Cross-sectional | Clinic | CA | Reverse Dot Blot | - | Anal | 98 |  |  | 95 (96.94%) | | | - | | - | | | Y | | Y | | N |
| 88 | Gao M et al.2019 | Eastern | Jan. 2017 - Dec. 2017 | Case-control | Clinic | CA | Flow-through Hybridization | - | mutiple sites | 720 |  |  | 458 (63.61%) | | | - | | - | | | Y | | N | | N |
| 89 | Li T et al.2012 | Western | Feb. 2009 - Mar. 2011 | Cross-sectional | Clinic | outpatients | Flow-through Hybridization | - | Anal | 58 |  |  | 31 (53.45%) | | | 22 (37.93%) | | 24 (41.38%) | | | Y | | Y | | N |
| 90 | Li Y et al.2017 | Eastern | Jan. 2014 - Dec. 2016 | Cross-sectional | hospital | MSM | FQ-PCR | - | Genital | 168 | Mean30.1(SD7.8) |  | 127 (75.60%) | | | 88 (52.38%) | | 106 (63.10%) | | | Y | | Y | | N |
| 91 | Liang H et al.2013 | Eastern | June 2011 - June 2012 | Cross-sectional | Hospital | CA | Flow-through Hybridization | - | Anal | 41 |  |  | 34 (82.93%) | | | 9 (21.95%) | | 25 (60.98%) | | | Y | | Y | | N |
| 92 | Liang X et al.2014 | Eastern | Feb. 2011 - Oct. 2013 | Cross-sectional | Hospital | common anus and rectal diseases | Gene chip | - | Genital | 364 |  | 10-80 | 117 (32.14%) | | | - | | - | | | Y | | Y | | N |
| 93 | Lin Q et al.2013 | Eastern | Nov. 2011 - Apr. 2011 | Cross-sectional | Hospital | CA | Gene chip | - | mutiple sites | 112 |  |  | 93 (83.04%) | | | 35 (31.25%) | | - | | | Y | | Y | | N |
| 94 | Min X et al.2017 | Western | Jan. 2012 - Mar. 2015 | Cross-sectional | Hospital | LSCC | Reverse Dot Blot | - | Head and neck | 44 |  |  | 20 (45.45%) | | | - | | - | | | Y | | N | | N |
| 95 | Pu J et al.2013 | Eastern | Jan. 2010 - Dec. 2012 | Cross-sectional | Hospital | CA | Gene chip | - | mutiple sites | 409 |  |  | 370 (90.46%) | | | - | | - | | | Y | | Y | | N |
| 96 | Sun X et al.2014 | Eastern | Jan. 2010 - Dec. 2012 | Case-control | Clinic | outpatients & health checkups | Reverse Dot Blot | - | Anal | 94 (56+38) | Mean38.5(SD16.5) | 22-60 | 26 (27.66%) | | | 21 (22.34%) | | 10 (10.64%) | | | Y | | Y | | N |
| 97 | Tian T et al.2017 | Western | Mar. 2016 - May 2016 | Cross-sectional | Clinic and community(MSM) | HIV Negative MSM | Flow-through Hybridization | - | Genital | 200 |  |  | 108 (54.00%) | | | 84 (42.00%) | | 51 (25.50%) | | | Y | | Y | | Y |
| 98 | Wang W et al.2013 | Eastern | June 2011 - Dec. 2011 | Cross-sectional | Hospital | CA | Membrane hybridization | - | Anal | 18 |  |  | 10 (55.56%) | | | 5 (27.78%) | | - | | | Y | | N | | N |
| 99 | Wang Z et al.2014 | Central | Jan. 2001 - Jan. 2011 | Cross-sectional | Hospital | SSCC | Unknown | - | mutiple sites | 196 |  |  | 130 (66.33%) | | | - | | - | | | Y | | N | | N |
| 100 | Wu Y et al.2020 | Eastern | Oct. 2017 - Oct. 2019 | Cross-sectional | Hospital | CA | Flow-FISH | - | Anal | 1247 |  |  | 492 (39.45%) | | | - | | - | | | Y | | Y | | N |
| 101 | Xu Y et al.2017 | Western | Jan. 2006 - Jan. 2016 | Cross-sectional | Hospital | SSCC | Unknown | - | mutiple sites | 201 |  |  | 134 (66.67%) | | | - | | - | | | Y | | N | | N |
| 102 | Yang A et al.2018 | Central | Jan. 2015 - Feb. 2017 | Cross-sectional | Clinic | CA | Reverse Dot Blot | - | mutiple sites | 165 |  |  | 149 (90.30%) | | | - | | - | | | Y | | Y | | N |
| 103 | Yang Q et al.2019 | Eastern | - | Cross-sectional | Clinic | health checkups | FQ-PCR | - | Anal | 473 |  |  | 13 (2.75%) | | | 12 (2.54%) | | 2 (0.42%) | | | Y | | Y | | Y |
| 104 | Yao Y et al.2021 | Eastern | Jan. 2019 - Jan. 2020 | Cross-sectional | Hospital | CA | Reverse Dot Blot | - | mutiple sites | 69 |  |  | 65 (94.20%) | | | 15 (21.74%) | | 50 (72.46%) | | | Y | | N | | N |
| 105 | Zhong W et al.2021 | Eastern | Jan. 2014 - July 2019 | Cross-sectional | Clinic | outpatients | Reverse Dot Blot | - | mutiple sites | 1038 | Mean33.6(SD11) | 17-83 | 706 (68.02%) | | | - | | - | | | Y | | Y | | Y |
| 106 | Zhong M et al.2016 | Eastern | Aug. 2014 - Apr. 2016 | Cross-sectional | STD clinic | outpatients | Flow-FISH | - | mutiple sites | 600 |  |  | 276 (46.00%) | | | 134 (22.33%) | | 210 (35.00%) | | | Y | | Y | | N |
| 107 | Zhu Z et al.2018 | Eastern | Jan. 2016 - May 2017 | Cross-sectional | Clinic | Spouses of HPV-infected females | Reverse Dot Blot | - | Anal | 165 |  |  | 64 (38.79%) | | | - | | - | | | Y | | Y | | N |
| 108 | Luo J et al.2019 | Eastern | Oct. 2017 - Apr. 2018 | Cross-sectional | Clinic | infertile male | FQ-PCR | - | Sperm | 436 | Mean31.4(SD5.3) | 22-45 | 28 (6.42%) | | | 16 (3.67%) | | 13 (2.98%) | | | Y | | Y | | N |
| 109 | Mo H et al.2015 | Eastern | Aug. 2012 - Sep. 2013 | Cross-sectional | Clinic | outpatients | Flow-through Hybridization | - | Anal | 350 | Mean31(SD11.2) | 18-63 | 131 (37.43%) | | | 44 (12.57%) | | 87 (24.86%) | | | Y | | Y | | Y |
| 110 | Muergan M et al.2013 | Western | June 2009 - July 2009 | Cross-sectional | Community | health checkups | Flow-through Hybridization | - | Anal | 430 |  |  | 113 (26.28%) | | | 101 (23.49%) | | 12 (2.79%) | | | Y | | Y | | N |
| 111 | Pan L et al.2018 | Eastern | Aug. 2016 - Dec. 2016 | Cross-sectional | Clinic | Spouses of HPV-infected females | Reverse Dot Blot | - | Anal | 139 | Mean35.7(SD8.5) | 18-63 | 116 (83.45%) | | | - | | - | | | Y | | Y | | Y |
| 112 | Qiao Y et al.2020 | Western | Feb. 2018 - May 2019 | Cross-sectional | Clinic | infertile male | Membrane hybridization | - | Sperm | 326 | Mean37.1(SD3.6) | 23-45 | 57 (17.48%) | | | 37 (11.35%) | | 20 (6.13%) | | | Y | | Y | | N |
| 113 | Ruan J et al.2012 | Eastern | Oct. 2008 - Aug. 2011 | Cross-sectional | Clinic | outpatients | Flow-through Hybridization | - | Anal | 1206 | Mean28.7(SD12) | 16-64 | 342 (28.36%) | | | 168 (13.93%) | | 174 (14.43%) | | | Y | | Y | | Y |
| 114 | Shen X et al.2013 | Western | Dec. 2010 - Sep. 2012 | Case-control | Clinic | CA/health checkups | FQ-PCR | - | mutiple sites | 63 | Mean31.2(SD13.5) | 19-67 | 38 (60.32%) | | | - | | - | | | Y | | N | | N |
| 115 | Sun W et al.2014 | Eastern | Jan. 2010 - Aug. 2012 | Cross-sectional | Hospital | CA | Flow-through Hybridization | - | Anal | 26 |  | 17-76 | 24 (92.31%) | | | - | | - | | | Y | | Y | | N |
| 116 | Tuo Y et al.2015 | Western | Jan. 2011 - Dec. 2013 | Cross-sectional | Hospital | infertile male | Membrane hybridization | - | Sperm | 330 |  | 20-40 | 30 (9.09%) | | | - | | - | | | Y | | Y | | N |
| 117 | Wan C et al.2018 | Eastern | Feb. 2016 - Feb. 2018 | Cross-sectional | Clinic | outpatients | Gene chip | - | Anal | 602 | Mean35(SD14.5) | 16-74 | 290 (48.17%) | | | 153 (25.42%) | | 206 (34.22%) | | | Y | | Y | | N |
| 118 | Wang M et al.2015 | Eastern | Jan. 2011 - Dec. 2013 | Cross-sectional | Clinic | outpatients | Gene chip | - | Anal | 102 | Mean34.3(SD10.5) | 21-68 | 58 (56.86%) | | | - | | - | | | Y | | Y | | N |
| 119 | Wang C et al.2013 | Eastern | June 2011 - Aug. 2012 | Cross-sectional | Clinic and community | MSM | Flow-FISH | - | Genital | 302 | Mean30.9(SD9.5) | 19-57 | 205 (67.88%) | | | 177 (58.61%) | | 140 (46.36%) | | | Y | | Y | | N |
| 120 | Qi C et al.2017 | Eastern | July 2014 - June 2016 | Cross-sectional | Hospital | CA | Unknown | - | mutiple sites | 307 | Mean32.3(SD11.3) | 17-85 | 281 (91.53%) | | | 134 (43.65%) | | 258 (84.04%) | | | Y | | Y | | Y |
| 121 | Wang G et al.2020 | Eastern | Jan. 2018 - Dec. 2018 | Cross-sectional | Clinic | outpatients | Membrane hybridization | - | Anal | 712 | Mean36(SD12.2) | 15-76 | 210 (29.49%) | | | - | | - | | | Y | | Y | | Y |
| 122 | Wei J et al.2022 | Eastern | Jan. 2016 - Dec. 2018 | Cross-sectional | Clinic | outpatients | Flow-through Hybridization | - | mutiple sites | 229 | Median35 | 18-81 | 70 (30.57%) | | | - | | - | | | Y | | Y | | Y |
| 123 | Wu Y et al.2015 | Eastern | Dec. 2012 - Dec. 2013 | Cross-sectional | Clinic | outpatients | Reverse Dot Blot | - | mutiple sites | 1060 |  |  | 417 (39.34%) | | | - | | - | | | Y | | N | | N |
| 124 | Yao X et al.2015 | Central | Jan. 2013 - Dec. 2014 | Cross-sectional | Clinic | CA | Reverse Dot Blot | - | mutiple sites | 120 | Mean35.2(SD11.2) | 16-61 | 83 (69.17%) | | | - | | - | | | Y | | Y | | N |
| 125 | Yu G et al.2018 | Eastern | Aug. 2012 - Aug. 2016 | Cross-sectional | Clinic | outpatients | FQ-PCR | - | mutiple sites | 3195 |  |  | 1643 (51.42%) | | | 790 (24.73%) | | 1263 (39.53%) | | | Y | | N | | N |
| 126 | Yu J et al.2016 | Eastern | Mar. 2012 - June 2014 | Cross-sectional | Clinic | outpatients | Gene chip | - | Anal | 2580 | Mean41(SD14.3) | 17-65 | 1164 (45.12%) | | | 570 (22.09%) | | 876 (33.95%) | | | Y | | Y | | N |
| 127 | Zhang G et al.2015 | Western | - | Cross-sectional | Hospital | CA | Flow-through Hybridization | - | mutiple sites | 46 |  |  | 45 (97.83%) | | | - | | - | | | Y | | N | | N |
| 128 | Zhang J et al.2017 | Mutiple sites | Aug. 1985 - July 2017 | Cross-sectional | Hospital | Suspected CA | Reverse Dot Blot | - | Genital | 179 | Mean42.2(SD8.9) |  | 136 (75.98%) | | | - | | - | | | Y | | Y | | Y |
| 129 | Zhang R et al.2018 | Eastern | - | Case-control | Rural community | Spouses of cervical lesion patients & health checkups | Gene chip | - | Anal | 398 (294+104) |  |  | 45 (11.31%) | | | 34 (8.54%) | | 14 (3.52%) | | | Y | | Y | | Y |
| 130 | Zhang R et al.2017 | Eastern | Jan. 2013 - June 2015 | Cross-sectional | Clinic | outpatients | FQ-PCR | - | Anal | 233 | Median33 | 19-83 | 42 (18.03%) | | | 33 (14.16%) | | 16 (6.87%) | | | Y | | Y | | N |
| 131 | Zhao X et al.2017 | Eastern | Jan. 2016 - Nov. 2016 | Cross-sectional | Clinic | Spouses of HPV-infected females | Flow-through Hybridization | - | Anal | 146 | Mean38.4(SD7.8) | 25-56 | 64 (43.84%) | | | 64 (43.84%) | | 0 (0.00%) | | | Y | | Y | | Y |
| 132 | Zheng Y et al.2021 | Eastern | Jan. 2009 - June 2018 | Cross-sectional | Clinic（male with HIV) | HIV Positive | Flow-through Hybridization | - | Genital | 201 | Mean35.1(SD11.1) |  | 190 (94.53%) | | | 103 (51.24%) | | 185 (92.04%) | | | Y | | Y | | N |
| 133 | Chen J et al.2020 | Eastern | July 2015 - June 2018 | Cross-sectional | Clinic | outpatients | Reverse Dot Blot | - | mutiple sites | 3865 | Mean34.6(SD10.6) |  | 2019 (52.24%) | | | - | | - | | | Y | | Y | | Y |
| 134 | Cheng Y et al.2017 | Eastern | Oct. 2013 - Jan. 2017 | Cross-sectional | Clinic | MSM with CA | Flow-FISH | - | mutiple sites | 162 | Median31 | 18-59 | 162 (100.00%) | | | 86 (53.09%) | | - | | | Y | | Y | | N |
| 135 | Cheng L et al.2021 | Eastern | Jan. 2016 - June 2019 | Cross-sectional | Clinic | outpatients | Flow-FISH | - | mutiple sites | 244 | Mean34(SD16.8) | 14-81 | 100 (40.98%) | | | 57 (23.36%) | | 56 (22.95%) | | | Y | | Y | | Y |
| 136 | Duan B et al.2021 | Central | Oct. 2016 - Oct. 2019 | Case-control | Hospital | CA | FQ-PCR | - | Genital | 160 |  |  | 160 (100.00%) | | | 100 (62.50%) | | - | | | Y | | N | | N |
| 137 | Fan J et al.2018 | Central | Jan. 2012 - Dec. 2016 | trial | AIDS center(male with HIV) | HIV Positive MSM | FQ-PCR | - | Genital | 77 |  |  | 70 (90.91%) | | | 59 (76.62%) | | - | | | Y | | Y | | N |
| 138 | Fang W et al.2013 | Western | June 2001 - June 2010 | Cross-sectional | Clinic | outpatients | Reverse Dot Blot | - | Anal | 552 | Mean31(SD6.7) | 20-42 | 157 (28.44%) | | | - | | - | | | Y | | N | | N |
| 139 | Fu Y et al.2020 | Northeastern | June 2017 - Dec. 2019 | Cross-sectional | Clinic | CA&health checkups | Reverse Dot Blot | - | Anal | 205(155+50) | Mean34.2(SD10.5) | 17-68 | 106 (51.71%) | | | - | | - | | | Y | | Y | | N |
| 140 | Gao Y et al.2019 | Central | Jan. 2014 - June 2016 | Cross-sectional | CDC | CA | FQ-PCR | - | mutiple sites | 73 |  |  | 62 (84.93%) | | | 18 (24.66%) | | 44 (60.27%) | | | Y | | N | | N |
| 141 | Han w et al.2017 | Western | Nov. 2015 - July 2016 | Cross-sectional | Clinic | outpatients | Reverse Dot Blot | - | Anal | 478 | Mean33(SD9.7) | <87 | 215 (44.98%) | | | 107 (22.38%) | | 192 (40.17%) | | | Y | | Y | | Y |
| 142 | He Y et al.2015 | Western | Sep. 2011 - Sep. 2013 | Cross-sectional | Hospital | Suspected CA | FQ-PCR | - | Anal | 59 |  |  | 12 (20.34%) | | | - | | - | | | Y | | N | | N |
| 143 | Hu F et al.2014 | Central | Jan. 2010 - Dec. 2012 | Cross-sectional | Hospital | CA | Gene chip | - | Genital | 154 |  |  | 80 (51.95%) | | | - | | - | | | Y | | Y | | N |
| 144 | Huang Z et al.2018 | Eastern | July 2012 - Sep. 2016 | Cross-sectional | Hospital | CA | Flow-through Hybridization | - | Anal | 500 | Median32 |  | 388 (77.60%) | | | - | | - | | | Y | | Y | | Y |
| 145 | Ji Z et al.2022 | Eastern | July 2013 - Dec. 2019 | Cross-sectional | Clinic | outpatients | Reverse Dot Blot | - | Anal | 5852 |  |  | 3107 (53.09%) | | | - | | - | | | Y | | Y | | N |
| 146 | Jiang L et al.2018 | Eastern | Jan. 2012 - Aug. 2016 | Cross-sectional | Hospital | Suspected CA | Reverse Dot Blot | GP5+/GP6+ | mutiple sites | 2830 | Mean33.6(SD10.8) | 10-84 | 1621 (57.28%) | | | - | | - | | | Y | | Y | | Y |
| 147 | Li J et al.2014 | Eastern | Jan. 2012 - Dec. 2012 | Cross-sectional | Clinic | outpatients | Reverse Dot Blot | - | Anal | 748 | Mean35.5(SD14.5) | 15-73 | 354 (47.33%) | | | - | | - | | | Y | | Y | | N |
| 148 | Li L et al.2012 | Eastern | Oct. 2008 - June 2010 | Cross-sectional | Clinic（MSM） | MSM with CA | Flow-through Hybridization | - | Genital | 57 | Mean32.5(SD12.3) | 16-65 | 56 (98.25%) | | | 20 (35.09%) | | 36 (63.16%) | | | Y | | N | | N |
| 149 | Liang M et al.2022 | Central | Aug. 2018 - Dec. 2020 | Cross-sectional | Hospital | outpatients | Reverse Dot Blot | - | mutiple sites | 742 | Mean37.1(SD11.8) |  | 376 (50.67%) | | | - | | - | | | Y | | Y | | Y |
| 150 | Liao N et al.2013 | Eastern | May 2010 - July 2010 | Cross-sectional | Clinic and community | MSM | Flow-FISH | - | Genital | 289 | Mean29.5(SD7.3) | 18-55 | 207 (71.63%) | | | 163 (56.40%) | | 98 (33.91%) | | | Y | | Y | | N |
| 151 | Liu M et al.2014 | Eastern | Jan. 2014 - Aug. 2014 | Cross-sectional | Clinic | outpatients | FQ-PCR | - | Anal | 330 | Mean33.1(SD9.4) |  | 157 (47.58%) | | | 101 (30.61%) | | 106 (32.12%) | | | Y | | Y | | N |
| 152 | Zhao Q et al.2016 | Western | Jan. 2014 - July 2015 | Cross-sectional | Clinic | outpatients | FQ-PCR | - | Anal | 121 |  |  | - | | | 60 (49.59%) | | - | | | N | | N | | N |
| 153 | Zhao X et al.2017 | Eastern | June 2014 - June 2016 | Cross-sectional | Clinic | CA | Flow-through Hybridization | - | Anal | 443 |  |  | 443 (100.00%) | | | - | | - | | | Y | | Y | | N |
| 154 | Zhao X et al.2016 | Eastern | July 2014 - June 2015 | Cross-sectional | Clinic | phimosis | Flow-through Hybridization | - | Anal | 70 | Mean35.5(SD10.8) | 18-56 | - | | | 32 (45.71%) | | - | | | N | | Y | | Y |
| 155 | Zhong Y et al.2018 | Eastern | May 2015 - May 2017 | Cross-sectional | Hospital | outpatients | Reverse Dot Blot | - | mutiple sites | 1147 | Mean31.1(SD8.2) |  | 669 (58.33%) | | | - | | - | | | Y | | Y | | Y |
| 156 | Zhu K et al.2018 | Northeastern | Sep. 2017 - Apr. 2018 | Cross-sectional | Hospital | outpatients | Reverse Dot Blot | - | Anal | 365 | Mean34.3(SD12) | 13-71 | 220 (60.27%) | | | - | | - | | | Y | | Y | | N |
| 157 | Wang G et al.2019 | Eastern | Jan. 2017 - July 2018 | Cross-sectional | Clinic | Spouses of HPV-infected females | Flow-FISH | - | Anal | 579 | Mean43.3(SD11) | 23-67 | 229 (39.55%) | | | - | | - | | | Y | | Y | | N |
| 158 | Wang J et al.2014 | Western | Apr. 2012 - Apr. 2013 | Cross-sectional | Clinic | outpatients | FQ-PCR | - | Anal | 552 | Mean35.3(SD10.5) | 23-65 | 214 (38.77%) | | | - | | - | | | Y | | Y | | Y |
| 159 | Wu M et al.2017 | Eastern | Aug. 2012 - Nov. 2016 | Cross-sectional | Clinic | Spouses of HPV-infected females | Flow-FISH | - | Anal | 202 | Mean35(SD13.5) | 16-70 | 93 (46.04%) | | | - | | - | | | Y | | Y | | Y |
| 160 | Wu R et al.2016 | Eastern | Aug. 2014 - Dec. 2015 | Cross-sectional | Clinic | CA | Flow-FISH | - | Anal | 404 | Mean36.7(SD10.2) | 16-67 | 164 (40.59%) | | | 91 (22.52%) | | 101 (25.00%) | | | Y | | Y | | Y |
| 161 | Wu Y et al.2012 | Eastern | June 2009 - Feb. 2011 | Cross-sectional | Hospital | CA | Flow-through Hybridization | - | Anal | 97 |  |  | 94 (96.91%) | | | 25 (25.77%) | | 85 (87.63%) | | | Y | | Y | | N |
| 162 | Wu Z et al.2019 | Eastern | June 2017 - May 2018 | Cross-sectional | Clinic | Spouses of HPV-infected females | Capture Hybridization | - | Anal | 87 |  |  | 38 (43.68%) | | | - | | - | | | Y | | N | | N |
| 163 | Xia S et al.2013 | Eastern | Jan. 2010 - Jan. 2013 | Cross-sectional | Clinic | CA | FQ-PCR | - | Anal | 304 | Mean32.6(SD12.7) | 18-69 | 117 (38.49%) | | | - | | - | | | Y | | Y | | Y |
| 164 | Xie L et al.2017 | Eastern | Dec. 2015 - May 2016 | Cross-sectional | Hospital | Spouses of HPV-infected females | Reverse Dot Blot | - | Anal | 110 | Mean41.3(SD9.7) | 21-63 | 37 (33.64%) | | | 28 (25.45%) | | 17 (15.45%) | | | Y | | Y | | N |
| 165 | Xu J et al.2018 | Eastern | Jan. 2014 - Dec. 2017 | Cross-sectional | Hospital | CA | FQ-PCR | - | mutiple sites | 80 |  |  | 80 (100.00%) | | | 23 (28.75%) | | 65 (81.25%) | | | Y | | Y | | N |
| 166 | Xu L et al.2018 | Eastern | May 2016 - Dec. 2016 | Cross-sectional | Clinic | Spouses of HPV-infected females | Reverse Dot Blot | - | Anal | 312 | Mean29.1(SD6.3) | 20-45 | 176 (56.41%) | | | - | | - | | | Y | | N | | N |
| 167 | Xu S et al.2019 | Eastern | Apr. 2014 - Aug. 2017 | Cross-sectional | Clinic | outpatients | Flow-through Hybridization | - | Anal | 835 | Median31 | 18-77 | 207 (24.79%) | | | 154 (18.44%) | | 85 (10.18%) | | | Y | | Y | | N |
| 168 | Xu J et al.2020 | Central | Jan. 2017 - Jan. 2018 | Cross-sectional | Clinic | Spouses of cervical lesion patients | FQ-PCR | - | Anal | 100 | Mean37.2(SD8.2) |  | 56 (56.00%) | | | - | | - | | | Y | | Y | | N |
| 169 | Yang H et al.2019 | Mutiple sites | Nov. 2018 - Apr. 2019 | Cross-sectional | Hospital（MSM） | MSM | Reverse Dot Blot | - | mutiple sites | 235 | Mean31.2(SD12.5) | 18-68 | - | | | - | | - | | | N | | N | | N |
| 170 | Yuan L et al.2019 | Central | June 2014 - June 2015 | Cross-sectional | Clinic | outpatients | Reverse Dot Blot | - | Head and neck | 495 | Mean33.9(SD16.5) |  | 115 (23.23%) | | | - | | - | | | Y | | Y | | Y |
| 171 | Zhang H et al.2016 | Eastern | Jan. 2010 - Dec. 2014 | Cross-sectional | Clinic | OPSCC | Reverse Dot Blot | - | Head and neck | 28 |  |  | 5 (17.86%) | | | - | | - | | | Y | | N | | N |
| 172 | Zhang J et al.2017 | Eastern | Apr. 2012 - Feb. 2014 | Cross-sectional | Clinic | outpatients | FQ-PCR | - | Anal | 650 |  | 18-80 | 148 (22.77%) | | | - | | - | | | Y | | Y | | N |
| 173 | Zhang J et al.2019 | Western | Oct. 2012 - May 2016 | Cross-sectional | Clinic | Spouses of HPV-infected females | Reverse Dot Blot | - | mutiple sites | 519 |  |  | 366 (70.52%) | | | 181 (34.87%) | | 311 (59.92%) | | | Y | | N | | N |
| 174 | Zhang N et al.2018 | Eastern | Jan. 2014 - Dec. 2017 | Cross-sectional | Clinic | CA | FQ-PCR | - | mutiple sites | 623 | Median32 |  | 473 (75.92%) | | | 201 (32.26%) | | 426 (68.38%) | | | Y | | N | | N |
| 175 | Zhang Q et al.2018 | Western | Sep. 2016 - May 2017 | Cross-sectional | Clinic | CA | Gene chip | - | mutiple sites | 224 |  |  | 203 (90.63%) | | | 69 (30.80%) | | 182 (81.25%) | | | Y | | Y | | N |
| 176 | Zhang S et al.2015 | Eastern | Oct. 2013 - Mar. 2015 | Cross-sectional | Clinic | MSM | Flow-FISH | - | Genital | 301 | Median32 | 18-65 | 237 (78.74%) | | | 152 (50.50%) | | 208 (69.10%) | | | Y | | Y | | N |
| 177 | Zhang X et al.2015 | Eastern | June 2012 - Dec. 2014 | Cross-sectional | Clinic | outpatients | Reverse Dot Blot | - | Genital | 55 | Mean31(SD9) | 18-60 | - | | | - | | - | | | N | | Y | | N |
| 178 | Zhang Y et al.2018 | Eastern | Jan. 2016 - Dec. 2017 | Cross-sectional | Clinic | CA | FQ-PCR | - | Anal | 582 | Mean33.5 | 17-65 | 228 (39.18%) | | | 140 (24.05%) | | 138 (23.71%) | | | Y | | Y | | N |
| 179 | Zhang J et al.2017 | Central | Jan. 2014 - June 2016 | Cross-sectional | Clinic | CA | Reverse Dot Blot | - | mutiple sites | 140 | Mean34.5(SD14.2) | 18-80 | 138 (98.57%) | | | 64 (45.71%) | | 106 (75.71%) | | | Y | | Y | | N |
| 180 | Li C et al.2020 | Eastern | May 2016 - Aug. 2019 | Cross-sectional | Clinic | Spouses of HPV-infected females | Reverse Dot Blot | - | Genital | 104 | Mean33.1(SD3.5) | 18-49 | 98 (94.23%) | | | - | | - | | | Y | | Y | | N |
| 181 | Li J et al.2019 | Eastern | Dec. 2015 - June 2017 | Cross-sectional | Clinic | outpatients | Reverse Dot Blot | - | Anal | 76 |  |  | 30 (39.47%) | | | 34 (44.74%) | | - | | | Y | | Y | | N |
| 182 | Li W et al.2016 | Eastern | Oct. 2011 - June 2015 | Cross-sectional | Clinic | CA | Gene chip | - | Anal | 68 | Mean33.6(SD11.5) | 16-62 | 67 (98.53%) | | | 23 (33.82%) | | 65 (95.59%) | | | Y | | Y | | N |
| 183 | Li X et al.2018 | Eastern | Jan. 2015 - Dec. 2016 | Case-control | Clinic | health checkups | FQ-PCR | - | Anal | 164 |  |  | 16 (9.76%) | | | 11 (6.71%) | | 5 (3.05%) | | | Y | | N | | N |
| 184 | Liu W et al.2014 | Eastern | Jan. 2009 - Jan. 2011 | Cross-sectional | Clinic | CA&health checkups | Reverse Dot Blot | - | Anal | 244 (184+60) |  |  | 64 (26.23%) | | | - | | - | | | Y | | N | | N |
| 185 | Liu X et al.2016 | Eastern | Jan. 2013 - June 2015 | Cross-sectional | Clinic | Spouses of HPV-infected females | Flow-through Hybridization | - | Anal | 120 | Mean37.6(SD7.3) | 26-55 | - | | | 52 (43.33%) | | - | | | N | | Y | | N |
| 186 | Liu Y et al.2017 | Eastern | Sep. 2014 - Aug. 2016 | Cross-sectional | Hospital | MSM with CA | Flow-through Hybridization | - | Genital | 116 | Mean29.4(SD6.9) | 18-52 | 82 (70.69%) | | | 52 (44.83%) | | 30 (25.86%) | | | Y | | Y | | N |
| 187 | Liu Y et al.2016 | Eastern | Oct. 2013 - May 2015 | Cross-sectional | Clinic | outpatients | FQ-PCR | - | Anal | 397 |  |  | 72 (18.14%) | | | 52 (13.10%) | | 37 (9.32%) | | | Y | | Y | | N |
| 188 | Long X et al.2013 | Eastern | Jan. 2010 - Apr. 2012 | Cross-sectional | Clinic | CA | Reverse Dot Blot | - | Anal | 176 | Mean32.1(SD10.3) | 20-61 | 110 (62.50%) | | | - | | - | | | Y | | Y | | Y |
| 189 | Luo Y et al.2016 | Eastern | Sep. 2014 - Sep. 2015 | Cross-sectional | Clinic | outpatients | Reverse Dot Blot | - | Anal | 214 | Mean34.7(SD14.8) | 11-70 | 98 (45.79%) | | | - | | - | | | Y | | Y | | N |
| 190 | Ma L et al.2012 | Western | June 2009 - June 2009 | Cross-sectional | Rural community | general population | Flow-through Hybridization | - | Anal | 430 |  |  | 113 (26.28%) | | | 101 (23.49%) | | 12 (2.79%) | | | Y | | Y | | N |
| 191 | Shang J et al.2020 | Northeastern | Mar. 2017 - Aug. 2019 | Cross-sectional | Clinic | outpatients | Reverse Dot Blot | - | mutiple sites | 155 |  | 18-68 | 73 (47.10%) | | | 55 (35.48%) | | 43 (27.74%) | | | Y | | Y | | Y |
| 192 | Shao L et al.2022 | Eastern | Jan. 2017 - Sep. 2019 | Cross-sectional | Hospital | outpatients | FQ-PCR | - | Anal | 1359 |  | 18-70 | 714 (52.54%) | | | 364 (26.78%) | | 523 (38.48%) | | | Y | | Y | | Y |
| 193 | Shi Y et al.2016 | Eastern | Jan. 2014 - Dec. 2014 | Cross-sectional | Clinic | outpatients | FQ-PCR | - | Anal | 607 | Mean39(SD12) | 17-67 | 295 (48.60%) | | | - | | - | | | Y | | Y | | N |
| 194 | Tan J et al.2012 | Eastern | May 2009 - July 2011 | Cross-sectional | Clinic | CA | Reverse Dot Blot | - | mutiple sites | 200 | Mean40(SD14.2) | 18-77 | 198 (99.00%) | | | - | | - | | | Y | | Y | | N |
| 195 | Tian C et al.2016 | Central | June 2013 - Mar. 2016 | Cross-sectional | Hospital | outpatients | Flow-through Hybridization | - | Anal | 117 | Mean36.7(SD11.5) | 17-81 | 69 (58.97%) | | | - | | - | | | Y | | Y | | N |
| 196 | Tong F et al.2018 | Central | June 2017 - Nov. 2017 | Cross-sectional | Hospital | outpatients | Reverse Dot Blot | - | Anal | 52 |  |  | 18 (34.62%) | | | - | | - | | | Y | | N | | N |
| 197 | Li P et al.2023 | Central | Jan. 2020 - Oct. 2022 | Cross-sectional | Clinic | outpatients | Flow-FISH | - | mutiple sites | 1303 |  |  | 673 (51.65%) | | | - | | - | | | Y | | N | | N |
| 198 | Wang T et al.2022 | Eastern | Jan. 2020 - Mar. 2021 | Cross-sectional | Clinic | outpatients | Flow-through Hybridization | - | Anal | 600 | Mean33.9(SD11.3) |  | 316 (52.67%) | | | - | | - | | | Y | | Y | | N |
| 199 | Hu W et al.2023 | Western | Dec. 2016 - Dec. 2018 | Cross-sectional | Hospital | CA | FQ-PCR | - | Anal | 499 |  | 15-80 | 398 (79.76%) | | | - | | - | | | Y | | N | | N |
| 200 | Wang C et al.2019 | Taiwan | Jan. 2013 - Dec. 2014 | Case-control | Hospital(male with HIV) | MSM | PCR-reverse blot hybridization | biotinylated | Genital | 496 | Mean32.9(SD7.8) |  | 279 (56.25%) | | | 218 (43.95%) | | 214 (43.15%) | | | Y | | N | | N |
| 201 | Lang B et al.2023 | Central | Aug. 2013 - Aug. 2019 | Cross-sectional | Hospital | PC | Capture Hybridization | - | Genital | 103 | Mean62(SD13.3) |  | 75 (72.82%) | | | - | | - | | | Y | | Y | | Y |
| 202 | Yuan H et al.2022 | Eastern | Aug. 2019 - June 2021 | Cross-sectional | Clinic | CA | Reverse Dot Blot | - | mutiple sites | 659 | Mean35.9 |  | 597 (90.59%) | | | 293 (44.46%) | | 543 (82.40%) | | | Y | | Y | | N |
| 203 | Zheng H et al.2023 | Central | Sep. 2017 - Aug. 2022 | Cross-sectional | Hospital | outpatients | Flow-through Hybridization | - | Anal | 5139 |  |  | 1739 (33.84%) | | | - | | - | | | Y | | Y | | N |
| 204 | Yang H et al.2023 | Eastern | Sep. 2011 - June 2012 | Cross-sectional | Clinic | outpatients | Flow-through Hybridization | - | Anal | 47 |  |  | 38 (80.85%) | | | 15 (31.91%) | | 32 (68.09%) | | | Y | | N | | N |
| 205 | Yuan H et al.2023 | Eastern | Aug. 2019 - June 2021 | Cross-sectional | Hospital | CA | Reverse Dot Blot | - | mutiple sites | 870 | Mean35.9 |  | 597 (68.62%) | | | 293 (33.68%) | | 543 (62.41%) | | | Y | | Y | | N |
| 206 | Jiang W et al.2022 | Western | Jan. 2019 - July 2021 | Cross-sectional | Clinic | outpatients | Reverse Dot Blot | - | mutiple sites | 301 | Mean32.1 | 15-85 | 235 (78.07%) | | | - | | - | | | Y | | Y | | Y |
| 207 | Zhang J et al.2022 | Eastern | Aug. 2016 - Oct. 2017 | Cross-sectional | VCT clinic(MSM) | MSM | Flow-through Hybridization | - | Genital | 414 | Mean31.1(SD10.3) |  | 248 (59.90%) | | | - | | - | | | Y | | N | | Y |
| 208 | Fan F et al.2023 | Central | Jan. 2020 - Jan. 2021 | Cross-sectional | Hospital | CA | FQ-PCR | - | mutiple sites | 119 |  | 18-55 | 100 (84.03%) | | | 55 (46.22%) | | 66 (55.46%) | | | Y | | Y | | N |
| 209 | Liang Y et al.2022 | Eastern | May 2021 - Sep. 2021 | Cross-sectional | Hospital（MSM） | HIV Negative MSM | Reverse Dot Blot | - | Genital | 100 | Median28 | 18-55 | 58 (58.00%) | | | 45 (45.00%) | | 30 (30.00%) | | | Y | | Y | | N |
| 210 | Nie L et al.2022 | Central | Jan. 2021 - Aug. 2021 | Cross-sectional | Clinic | outpatients | Unknown | - | Anal | 443 |  | 19-90 | 166 (37.47%) | | | 136 (30.70%) | | 111 (25.06%) | | | Y | | Y | | N |
| 211 | Qing Z et al.2020 | Eastern | Jan. 2018 - June 2019 | Cross-sectional | Hospital | outpatients | Flow-through Hybridization | - | Anal | 1077 | Mean46.7(SD9.5) | 22-68 | 402 (37.33%) | | | - | | - | | | Y | | Y | | N |
| 212 | Rao J et al.2022 | Western | Jan. 2015 - Jan. 2022 | Cross-sectional | Hospital | CA | FQ-PCR | - | Genital | 95 |  | 14-73 | 90 (94.74%) | | | 48 (50.53%) | | 70 (73.68%) | | | Y | | Y | | N |
| 213 | Song Y et al.2022 | Eastern | Oct. 2019 - May 2021 | Cross-sectional | Clinic | outpatients | Reverse Dot Blot | - | Anal | 1310 |  |  | 689 (52.60%) | | | - | | - | | | Y | | Y | | N |
| 214 | Sun L et al.2022 | Central | Jan. 2017 - Dec. 2021 | Cross-sectional | Clinic | outpatients | Flow-FISH | - | Anal | 557 |  | 17-86 | 239 (42.91%) | | | 129 (23.16%) | | 167 (29.98%) | | | Y | | Y | | Y |
| 215 | Wang J et al.2023 | Eastern | Jan. 2018 - Jan. 2020 | Cross-sectional | Hospital | CA | Reverse Dot Blot | - | Anal | 167 |  |  | 167 (100.00%) | | | - | | - | | | Y | | Y | | N |
| 216 | Lam E et al.2016 | Hong kong and Macau | Jan. 2005 - Dec. 2009 | Cross-sectional | Hospital | OPSCC | FQ-PCR | GP5t/GP6t_52HK | Head and neck | 178 |  |  | 34 (19.10%) | | | - | | - | | | Y | | N | | N |
| 217 | Qi Z et al.2013 | Central | Jan. 2009 - Dec. 2013 | Case-control | Hospital | ESCC & health checkups | Unknown | - | mutiple sites | 374 (189+185) |  |  | 116 (31.02%) | | | - | | - | | | Y | | N | | N |
| 218 | Zhang F et al.2022 | Eastern | Jan. 2019 - Dec. 2021 | Cross-sectional | Hospital | Spouses of HPV-infected females&health checkups | Unknown | - | Anal | 341(260+81) |  | 18-76 | 246 (72.14%) | | | - | | - | | | Y | | Y | | Y |
| 219 | Zhang X et al.2022 | Eastern | Dec. 2015 - Dec. 2020 | Cross-sectional | Clinic | outpatients | Reverse Dot Blot | - | Anal | 776 | Mean35(SD10.8) | 18-81 | 499 (64.30%) | | | 316 (40.72%) | | 405 (52.19%) | | | Y | | Y | | Y |
| 220 | Zhang Y et al.2016 | Eastern | Jan. 2009 - Dec. 2014 | Cross-sectional | Clinic | lichen sclerosus | FQ-PCR | - | Genital | 77 |  |  | 0 (0.00%) | | | - | | - | | | Y | | N | | N |
| 221 | Han Y et al.2022 | Northeastern | Dec. 2016 - Dec. 2019 | Cross-sectional | Clinic | outpatients | FQ-PCR | - | mutiple sites | 620 | Mean44.8(SD16) | 20-66 | 278 (44.84%) | | | - | | - | | | Y | | Y | | N |
| 222 | Ye Z et al.2022 | Northeastern | July 2020 - Dec. 2020 | Cross-sectional | VCT clinic(MSM) | MSM | Unknown | - | mutiple sites | 177 | Median29 |  | 135 (76.27%) | | | - | | - | | | Y | | N | | N |
| 223 | Ma Y et al.2021 | Eastern | Jan. 2019 - Dec. 2020 | Cross-sectional | Clinic | outpatients | FQ-PCR | - | Anal | 1162 | Median34 | 18-67 | 415 (35.71%) | | | 241 (20.74%) | | - | | | Y | | Y | | N |
| 224 | Zheng H et al.2014 | Western | Jan. 2011 - Dec. 2012 | Cross-sectional | Clinic | infertile male | Membrane hybridization | - | Sperm | 330 |  | 20-40 | 30 (9.09%) | | | - | | - | | | Y | | Y | | N |
| 225 | Zhao X et al.2018 | Eastern | May 2016 - Apr. 2017 | Cross-sectional | Clinic | health checkups | Flow-through Hybridization | - | Sperm | 150 | Mean32.3 | 25-40 | 22 (14.67%) | | | - | | - | | | Y | | Y | | N |
| 226 | Hu J et al.2022 | Eastern | June 2014 - June 2020 | Cohort | Clinic | outpatients | Flow-FISH | - | Anal | 2359 | Median38 | 15-82 | 928 (39.34%) | | | - | | - | | | Y | | Y | | N |
| 227 | Chang H et al. 2023 | Taiwan | Feb. 2018 - Dec. 2020 | Case-control | Hospital | Prostate cancer&benign prostatic hyperplasia | Unknown | - | mutiple sites | 278 |  |  | 12(4.32%) | | | - | | - | | | Y | | N | | N |
| 228 | Hou Y et al. 2023 | Eastern | Jan. 2017 - Dec. 2021 | Cross-sectional | Hospital | outpatients | Geno Array HPV | - | Anal | 756 |  |  | 144(19.05%) | | | - | | - | | | Y | | Y | | N |
| 229 | Lang B et al. 2023 | Central | - | Cross-sectional | Hospital | Prostate cancer | FQ-PCR | - | Anal | 59 | Mean72.8(SD6.2) |  | 10(16.95%) | | | - | | - | | | Y | | N | | N |
| 230 | Liu J et al. 2023 | Eastern | Jan. 2019 - Oct. 2021 | Cross-sectional | Hospital | CA | FQ-PCR | - | Genital | 549 | Mean31.5(SD) | 18-72 | 531(96.72%) | | | 389(70.86%) | | 508(92.53%) | | | Y | | N | | N |
| 231 | Yi J et al. 2023 | Eastern | Jan. 2017 - Dec. 2022 | Cross-sectional | Hospital | outpatients | Unknown | - | Anal | 4297 |  |  | 1071(24.92%) | | | - | | - | | | Y | | Y | | N |
| 232 | Zhang J et al. 2023 | Eastern | Aug. 2016 - June 2019 | Cross-sectional | CDC | HIV positive | Geno Array HPV | - | Genital | 772 |  | 18-82 | 374(48.45%) | | | 311(40.28%) | | 188(24.35%) | | | Y | | Y | | N |
| 233 | Zhou X et al. 2023 | Taiwan | Oct. 2015 - June 2019 | Cohort | community | MSM | Geno Array HPV | - | Genital | 201 | median27 IQR(24-32) | 65(32.34%) | | | 47(23.38%) | | 51(25.37%) | | Y | Y | | N | |  |  |
| 234 | Zhu Y et al. 2023 | Eastern | 2021 - | Cross-sectional | Population | general population | Unknown | - | Head and neck | 3048 | Mean56.6(SD12.9) | 20-80 | 112(3.67%) | | | 51(1.67%) | | 61(2.00%) | | | Y | | N | | N |
| 235 | Cui Y et al. 2023 | Eastern | July 2020 - Apr. 2023 | Cross-sectional | Hospital | Spouses of HPV-infected females | Flow-through Hybridization | - | Anal | 312 |  | 23-65 | 129(41.35%) | | | - | | - | | | Y | | Y | | Y |
| 236 | Fan J et al. 2018 | Central | June 2012 - Dec. 2013 | Cross-sectional | Hospital | HIV positive/negative MSM | Reverse Dot Blot | - | Genital | 131 |  |  | 106(80.92%) | | | 76(58.02%) | | 29(22.14%) | | | Y | | N | | N |
| 237 | Fang X et al. 2015 | Eastern | Apr. 2012 - Mar. 2014 | Cross-sectional | Hospital | outpatients | Reverse Dot Blot | - | mutiple sites | 1420 |  | 15-84 | 954(67.18%) | | | 496(34.93%) | | 817(57.54%) | | | Y | | Y | | Y |
| 238 | Gao L et al. 2020 | Eastern | Jan. 2013 - Dec. 2015 | Case-control | Hospital | laryngeal cancer & vocal fold polyp | FQ-PCR | - | Head and neck | 429 |  |  | 18(4.20%) | | | - | | - | | | Y | | N | | N |
| 239 | Han T et al. 2013 | Eastern | - | Cross-sectional | Hospital | CA & Health checkups | Reverse Dot Blot | - | Anal | 73 |  |  | 6(8.22%) | | | - | | - | | | Y | | Y | | N |
| 240 | Han T et al. 2015 | Eastern | Jan. 2013 - June 2014 | Cross-sectional | Hospital & VCT | HIV positive/negative MSM | Gene chip | - | Genital | 108 | Mean27.6(SD6.4) | 17-52 | 98(90.74%) | | | 49(45.37%) | | 15(13.89%) | | | Y | | Y | | N |
| 241 | Han Z et al. 2016 | Eastern | Jan. 2014 - Dec. 2015 | Cross-sectional | Hospital | outpatients | FQ-PCR | - | Anal | 2102 | Mean30.5(SD) |  | 88(4.19%) | | | - | | - | | | Y | | N | | N |
| 242 | Huang L et al. 2018 | Eastern | Jan. 2016 - Aug. 2017 | Cross-sectional | Hospital | outpatients | Flow-FISH | - | Anal | 351 |  |  | 135(38.46%) | | | 67(19.09%) | | 88(25.07%) | | | Y | | N | | N |
| 243 | Li F et al. 2023 | Eastern | Jan. 2012 - Dec. 2022 | Cross-sectional | Hospital | outpatients | FQ-PCR | - | Anal | 5078 |  | 16-89 | 1737(34.21%) | | | - | | - | | | Y | | Y | | Y |
| 244 | Li L et al. 2017 | Eastern | June 2012 - June 2015 | Cross-sectional | Hospital | MSM | Flow-through Hybridization | - | Genital | 132 | Mean28.9(SD9.9) | 17-57 | 132(100.00%) | | | - | | - | | | Y | | Y | | N |
| 245 | Li L et al. 2022 | Eastern | Jan. 2017 - June 2021 | Cross-sectional | Community and social media | HIV Negative MSM | ddPCR | - | Genital | 758 | Mean33.8(SD9.1) |  | 357(47.10%) | | | - | | - | | | Y | | Y | | N |
| 246 | Li Y et al. 2023 | Eastern | May 2021 - Oct. 2022 | Cross-sectional | Hospital | outpatients | FQ-PCR | - | mutiple sites | 820 | Mean34.5(SD10.3) | 18-70 | 239(29.15%) | | | - | | - | | | Y | | Y | | Y |
| 247 | Lin R et al. 2015 | Eastern | June 2011 - Dec. 2013 | Cross-sectional | Hospital | Spouses of HPV-infected females | Reverse Dot Blot | - | Sperm | 358 |  | 16-66 | 296(82.68%) | | | - | | - | | | Y | | N | | N |
| 248 | Liu Y et al. 2023 | Eastern | July 2018 - Dec. 2022 | Cross-sectional | Hospital | outpatients | Reverse Dot Blot | - | Anal | 258 | Mean34(SD9.3) | 20-67 | 103(39.92%) | | | 82(31.78%) | | 43(16.67%) | | | Y | | Y | | Y |
| 249 | Liu Z et al. 2018 | Western | Jan. 2017 - Dec. 2017 | Cross-sectional | Hospital | health checkups | FQ-PCR | - | Anal | 2773 | Mean32.2(SD5.7) | 20-57 | 37(1.33%) | | | - | | - | | | Y | | N | | Y |
| 250 | Long Y et al. 2014 | Eastern | Sep. 2012 - Apr. 2014 | Cross-sectional | Hospital | outpatients | FQ-PCR | - | Anal | 54 | Mean26.5(SD1.2) |  | 5(9.26%) | | | - | | - | | | Y | | N | | N |
| 251 | Na Z et al. 2023 | Eastern | Jan. 2018 - Dec. 2022 | Cross-sectional | Hospital | outpatients | Reverse Dot Blot | - | Anal | 1658 | Mean34.5(SD8.5) | 18-71 | 373(22.50%) | | | - | | - | | | Y | | Y | | Y |
| 252 | Shi Z et al. 2012 | Western | July 2008 - Dec. 2010 | Cross-sectional | Hospital | outpatients | FQ-PCR | - | Anal | 46 |  |  | 32(69.57%) | | | - | | - | | | Y | | N | | N |
| 253 | Sun X et al. 2023 | Central | Oct. 2019 - Feb. 2022 | Cross-sectional | Hospital | outpatients | FQ-PCR | - | mutiple sites | 1750 | Mean32.7(SD) | 15-76 | 582(33.26%) | | | - | | - | | | Y | | Y | | Y |
| 254 | Tang X et al. 2017 | Western | Nov. 2014 - Nov. 2016 | Cross-sectional | Hospital | Colonoscopy population | Gene chip | - | Genital | 156 |  |  | 5(3.21%) | | | - | | - | | | Y | | N | | Y |
| 255 | Wang D et al. 2023 | Eastern | Oct. 2021 - Oct. 2022 | Cross-sectional | Hospital | outpatients | FQ-PCR | - | Anal | 1415 | Mean35.5(SD10.3) |  | 566(40.00%) | | | - | | - | | | Y | | Y | | N |
| 256 | Wang H et al. 2016 | Eastern | Jan. 2015 - Dec. 2015 | Cross-sectional | Hospital | CA | Flow-FISH | - | mutiple sites | 47 |  |  | 40(85.11%) | | | - | | - | | | Y | | N | | N |
| 257 | Wang J et al. 2018 | Eastern | Feb. 2014 - Dec. 2015 | Cross-sectional | Hospital | CA | Unknown | - | Others | 57 | Mean33.4(SD) | 18-64 | 17(29.82%) | | | - | | - | | | Y | | N | | N |
| 258 | Wang J et al. 2020 | Eastern | Jan. 2016 - Jan. 2018 | Cross-sectional | Hospital | outpatients | ddPCR | MY09/11 | mutiple sites | 167 | Mean32.6(SD8.9) |  | 141(84.43%) | | | - | | - | | | Y | | N | | N |
| 259 | Wang M et al. 2023 | Eastern | Apr. 2021 - Apr. 2023 | Cross-sectional | Hospital | outpatients | Reverse Dot Blot | - | Anal | 4803 |  |  | - | | | - | | - | | | N | | Y | | N |
| 260 | Wang R et al. 2020 | Northeastern | Sep. 2017 - Dec. 2019 | Case-control | Hospital | Outpatients & health checkups | Flow-through Hybridization | - | Anal | 44 |  |  | 3(6.82%) | | | - | | - | | | Y | | N | | N |
| 261 | Wang T et al. 2022 | Western | Feb. 2015 - Oct. 2020 | Case-control | Hospital | outpatients | Gene chip | - | Anal | 208 |  | 22-44 | 115(55.29%) | | | - | | - | | | Y | | N | | N |
| 262 | Wei F et al. 2018 | Western | May 2014 - July 2014 | Cross-sectional | Population | general population | Unknown | GP 5+/6+ | Anal | 554 | median28 IQR(24-35) | 18-55 | 41(7.40%) | | | - | | - | | | Y | | Y | | N |
| 263 | Wu M et al. 2020 | Eastern | Jan. 2016 - Dec. 2018 | Cross-sectional | Hospital | outpatients | Reverse Dot Blot | - | mutiple sites | 675 |  |  | 327(48.44%) | | | - | | - | | | Y | | N | | Y |
| 264 | Xie Q et al. 2022 | Northeastern | Jan. 2018 - Jan. 2021 | Cross-sectional | Hospital | infertile male | ddPCR | - | Anal | 1921 |  | 18-50 | 78(4.06%) | | | - | | - | | | Y | | N | | Y |
| 265 | Xu Z et al. 2017 | Central | Nov. 2008 - Nov. 2015 | Cross-sectional | Hospital | Suspected CA/ CA | Reverse Dot Blot | - | Genital | 179 |  | 16-79 | 167(93.30%) | | | - | | - | | | Y | | N | | N |
| 266 | Yang S et al. 2018 | Central | Dec. 2016 - Dec. 2017 | Cross-sectional | Hospital | outpatients | FQ-PCR | - | Anal | 78 | Mean28.4(SD4.8) | 19-50 | 1(1.28%) | | | - | | - | | | Y | | N | | N |
| 267 | Yang X et al. 2015 | Western | - | Case-control | Hospital | infertile male & health checkups | Reverse Dot Blot | - | Sperm | 157 |  |  | 107(68.15%) | | | - | | - | | | Y | | Y | | N |
| 268 | Yang Y et al. 2016 | Eastern | - | Case-control | Hospital | infertile male & health checkups | Unknown | - | Sperm | 638 |  | 21-48 | 81(12.70%) | | | - | | - | | | Y | | N | | N |
| 269 | Zhang D et al. 2014 | Eastern | July 2009 - Sep. 2009 | Cross-sectional | STD clinic | MSM | Flow-through Hybridization | - | Genital | 404 | Mean30.3(SD7.4) | 18-64 | 147(36.39%) | | | - | | - | | | Y | | Y | | N |
| 270 | Zhang J et al. 2013 | Eastern | Jan. 1985 - May 2012 | Cross-sectional | Hospital | CA | ddPCR | - | Genital | 126 |  |  | 89(70.63%) | | | - | | - | | | Y | | N | | Y |
| 271 | Zhang J et al. 2015 | Eastern | July 2013 - June 2014 | Cross-sectional | Hospital | outpatients | FQ-PCR | - | Anal | 3039 |  |  | 103(3.39%) | | | - | | - | | | Y | | N | | N |
| 272 | Zhang L et al. 2020 | Eastern | Jan. 2018 - Dec. 2018 | Cross-sectional | Hospital | health checkups | Unknown | - | Anal | 873 |  |  | 68(7.79%) | | | - | | - | | | Y | | N | | N |
| 273 | Zhang T et al. 2023 | Western | Sep. 2018 - Dec. 2021 | Cross-sectional | Hospital | outpatients | FQ-PCR | - | Anal | 673 | Mean33.4(SD11) | 18-73 | 268(39.82%) | | | - | | - | | | Y | | Y | | N |
| 274 | Zou C et al. 2016 | Northeastern | Jan. 2014 - Dec. 2015 | Cross-sectional | Hospital | CA | FQ-PCR | - | Anal | 70 | Mean33.6(SD2.9) | 20-68 | 31(44.29%) | | | - | | - | | | Y | | N | | N |
| 275 | Zheng W et al. 2024 | Eastern China | Jan. 2020 - Dec. 2022 | Cross-sectional study | Clinic | outpatients | Reverse Dot Blot | - | Others | 1137 |  | 17-86 | 441(38.79%) | | | - | |  | | | Y | | Y- | | Y |
| 276 | Tian L et al. 2024 | Central China | May 2023 - Dec. 2023 | Cross-sectional study | Hospital | infertile male | FQ-PCR | - | Sperm | 115 | Mean38.5(SD10.2) |  | 11(9.57%) | | | - | | - | | | Y | | N | | N |
| 277 | Shen P et al. 20224 | Eastern China | Sep. 2018 - Sep. 2022 | Cross-sectional study | Clinic | outpatients | Unknown | - | Genital | 889 | Mean34.2(SD8.6) | 17-69 | 238(26.77%) | | | 158(17.77%) | | 117(13.16%) | | | Y | | Y | | Y |
| 278 | Na Z et al. 2024 | Eastern China | Jan. 2018 - Dec. 2022 | Cross-sectional study | Clinic | outpatients | FQ-PCR | - | Genital | 1658 | Mean34.4(SD8.5) | 17-71 | 373(22.50%) | | | - | | - | | | Y | | Y | | Y |
| 279 | Meng Y et al. 2024 | Eastern China | Jan. 2021 - Jun. 2023 | Cross-sectional study | Hospital | CA | FQ-PCR | - | Genital | 724 |  |  | 703(97.10%) | | | 366(50.55%) | | 583(80.52%) | | | Y | | Y | | N |
| 280 | Lv Z et al. 2024 | Western China | Apr. 2016 - Mar. 2023 | Cross-sectional study | Hospital (MSM) | MSM | Unknown | - | Anal | 1283 | Mean30.5(SD8.3) |  | 625(48.71%) | | | 464(36.17%) | | 161(12.55%) | | | Y | | N | | N |
| 281 | Lv Y et al. 2024 | Central China | Jun. 2020 - Nov. 2022 | Cross-sectional study | Hospital | outpatients | Unknown | - | Genital | 679 |  |  | 157(23.12%) | | | - | | - | | | Y | | Y | | Y |
| 282 | Lan Q et al. 2024 | Eastern China | Jun. 2021 - Mar. 2023 | Cross-sectional study | Clinic | outpatients | FQ-PCR | - | Genital | 1538 | Mean38.8(SD11.5) | 18-88 | 400(26.01%) | | | 321(20.87%) | | 127(8.26%) | | | Y | | N | | N |
| 283 | Hu J et al. 2024 | Western China | Jan. 2017 - Dec. 2022 | Cross-sectional study | Clinic | outpatients | FQ-PCR | - | Others | 1820 | Mean33.4(SD12.8) | 13-84 | 935(51.37%) | | | 240(13.19%) | | 872(47.91%) | | | Y | | N | | N |
| 284 | He P et al. 2024 | Eastern China | Jan. 2020 - Dec. 2022 | Cross-sectional study | Clinic | outpatients | FQ-PCR | - | Others | 7024 | Mean33.6(SD8.7) |  | 2793(39.76%) | | | - | | - | | | Y | | N | | N |
| 285 | Chen B et al. 2024 | Eastern China | Jan. 2018 - Jan. 2023 | Cross-sectional study | Clinic and Hospital | outpatients | FQ-PCR | - | Genital | 8432 |  | 2-91 | 3625(42.99%) | | | 3296(39.09%) | | 3203(37.99%) | | | Y | | Y | | Y |
| 286 | Zheng L et al. 2024 | Eastern China | Jan. 2016 - Dec. 2022 | Cross-sectional study | Clinic | outpatients | FQ-PCR | - | Genital | 3681 | Mean34.2(SD12.5) |  | 2388(64.87%) | | | 1564(42.49%) | | 2566(69.71%) | | | Y | | Y | | N |
| 287 | Zhang J et al. 2024 | Eastern China | Aug. 2016 - Jun. 2019 | Cohort study | Clinic(male with HIV) | HIV positive | Geno Array HPV | - | Anal | 772 |  | 18-82 | 374(48.45%) | | | 311(40.28%) | | 188(24.35%) | | | Y | | Y | | N |
| 288 | Zhang J et al. 2024 | Central China | Jan. 2022 - Apr. 2022 | Trial | Population | general population | Unknown | - | Others | 394 | Mean30.5(SD7.1) | 18-45 | 91(23.10%) | | | 71(18.02%) | | 38(9.64%) | | | Y | | N | | N |
| 289 | Yi X et al. 2024 | Western China | Jan. 2017 - Dec. 2022 | Cross-sectional study | Population | general population | Capture Hybridization | - | Others | 4831 |  |  | 651(13.48%) | | | 587(12.15%) | | 104(2.15%) | | | Y | | N | | N |
| 290 | Wang S et al. 2024 | Eastern China | Nov. 2015 - Oct. 2023 | Cross-sectional study | Clinic | outpatients | FQ-PCR | - | Others | 5114 |  |  | 1288(25.19%) | | | - | | - | | | Y | | N | | N |
| 291 | Wang J et al. 2024 | Eastern China | Jan. 2016 - Dec. 2022 | Cross-sectional study | Clinic | outpatients | Unknown | - | Genital | 591 | Mean35.5(SD9.9) | 3-84 | 68(11.51%) | | | - | | - | | | Y | | N | | N |
| 292 | Tian J et al. 2024 | Eastern China | Jan. 2019 - Nov. 2023 | Cross-sectional study | Clinic | outpatients | Reverse Dot Blot | - | Genital | 3900 |  | 11-83 | 1712(43.90%） | | | - | | - | | | Y | | N | | Y |
| 293 | Liu P et al. 2024 | Eastern China | Jan. 2012 - Aug. 2023 | Cross-sectional study | Hospital | outpatients | Geno Array HPV | - | Genital | 3737 |  | 27-37 | 1575(42.15%) | | | 856(22.91%) | | 1076(28.79%) | | | Y | | Y | | N |
| 294 | Huang Z et al. 2024 | Eastern China | Jan. 2015 - Dec. 2022 | Cross-sectional study | Hospital | outpatients | HybriMax | - | Others | 781 |  | 15-87 | 599(76.70%) | | | 162(20.74%) | | 559(71.57%) | | | Y | | Y | | N |
| 295 | Huang Y et al. 2024 | Eastern China | - | Cohort study | Hospital | Spouses of HPV-infected females | Reverse Dot Blot | - | Genital | 251 |  |  | 106(42.23%) | | | 81(32.27%) | | 57(22.71%) | | | Y | | Y | | N |
| 296 | Fan X et al. 2024 | Western China | Mar. 2023 - May 2023 | Cross-sectional study | Hospital | infertile male | FQ-PCR | - | Sperm | 1951 | Mean32.9(SD5.7) |  | 242(12.40%) | | | - | | - | | | Y | | N | | Y |

***Appendix6: Results of funnel plot, egger’s test, and trim and fill analysis***

| Publication bias | Health checkups | Outpatients |
| --- | --- | --- |
| egger’s test (*P*) | 0.634 | <0.001 |
| Funnel plot | 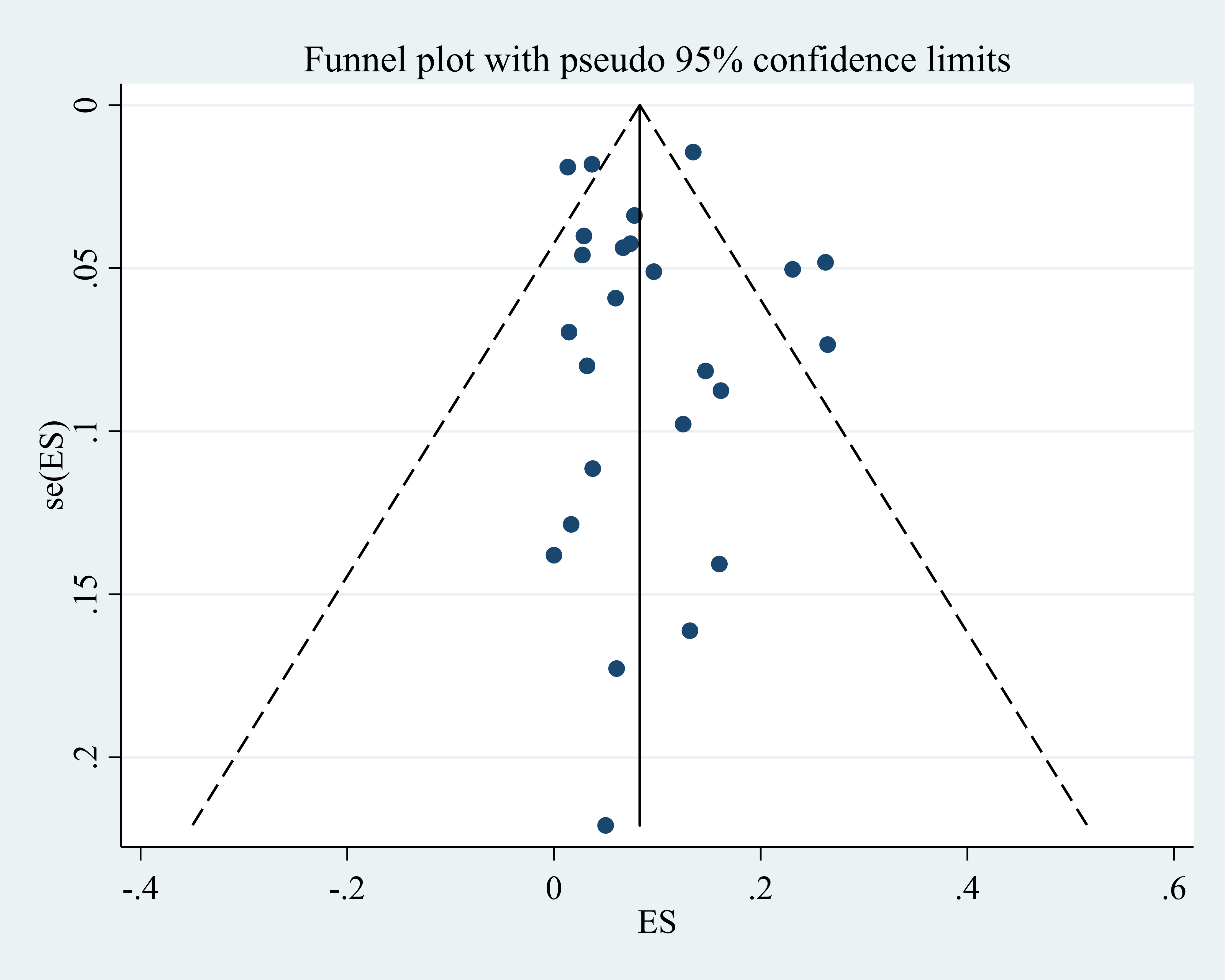 | 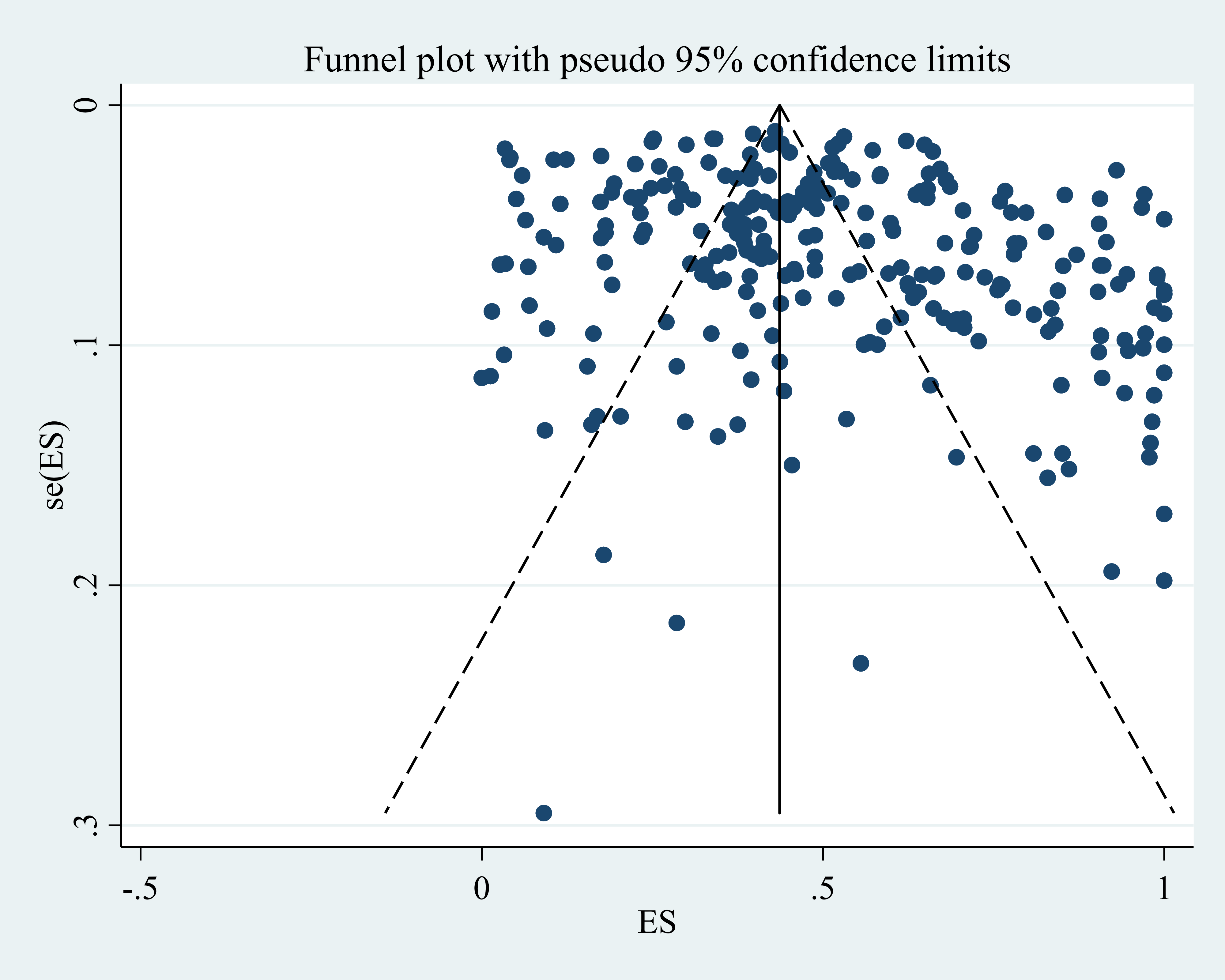 |

**The results of trim and fill analysis among outpatients：**

Meta-analysis

| Pooled 95% CI Asymptotic No. of

Method | Est Lower Upper z_value p_value studies

-------+----------------------------------------------------

Fixed | 0.436 0.432 0.441 191.956 0.000 273

Random | 0.509 0.484 0.535 39.024 0.000

Test for heterogeneity: Q= 8175.412 on 272 degrees of freedom (p= 0.000)

Moment-based estimate of between studies variance = 0.041

Trimming estimator: Linear

Meta-analysis type: Fixed-effects model

iteration | estimate Tn # to trim diff

----------+--------------------------------------

1 | 0.436 24048 39 37401

2 | 0.414 25687 51 3278

3 | 0.409 26055 54 736

4 | 0.406 26223 55 336

5 | 0.406 26273 56 100

6 | 0.406 26288 56 30

7 | 0.406 26288 56 0

Filled

Meta-analysis

| Pooled 95% CI Asymptotic No. of

Method | Est Lower Upper z_value p_value studies

-------+----------------------------------------------------

Fixed | 0.406 0.401 0.410 183.763 0.000 329

Random | 0.412 0.385 0.438 30.351 0.000

Test for heterogeneity: Q= 1.1e+04 on 328 degrees of freedom (p= 0.000)

Moment-based estimate of between studies variance = 0.055


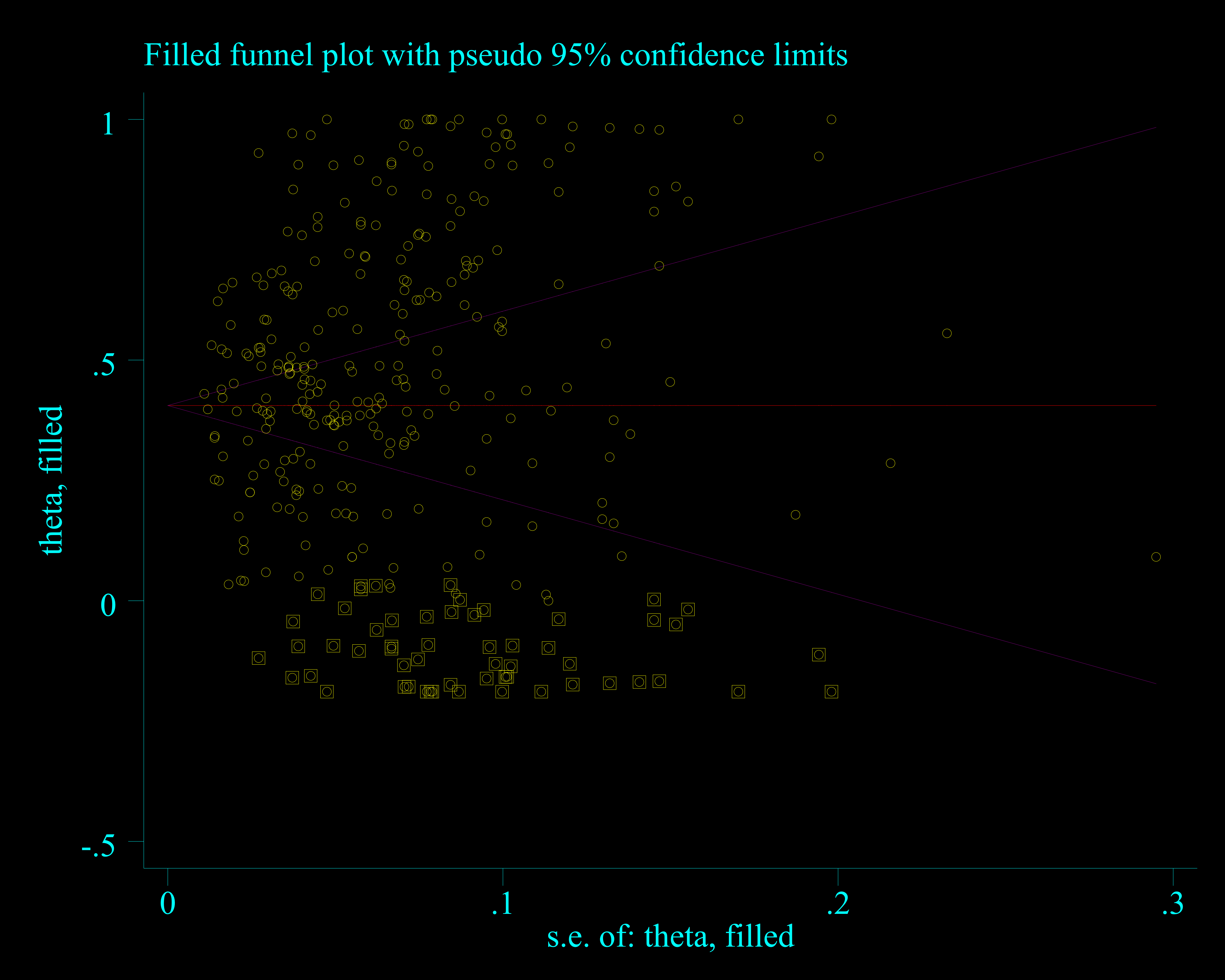


The filled funnel plot of outpatients

***Appendix7: Pooled prevalence of specific HPV genotypes among MSM and non-MSM***

| HPV genotypes | Number of studies | Number of males tested | Number of male HPV positive | Pooled prevalence of HPV positivity (95%CI) | *I*^2^ for heterogeneity | Number of studies | Number of males tested | Number of male HPV positive | | Pooled prevalence of HPV positivity (95%CI) | *I*^2^ for heterogeneity |  |  |
| --- | --- | --- | --- | --- | --- | --- | --- | --- | --- | --- | --- | --- | --- |
| HR genotypes | *MSM* | | | | |  | *Non-MSM* | | | | | | |
| HPV 16 | 41 | 8633 | 982 | 12.67(10.58,14.90) | 87.70 | 146 | 123944 | 8293 | | 7.35(6.54,8.20) | 96.54 |  |  |
| HPV 18 | 39 | 7573 | 587 | 7.30(5.44,9.38) | 89.54 | 128 | 112226 | 3250 | | 2.92(2.51,3.36) | 92.47 |  |  |
| HPV 31 | 31 | 6298 | 178 | 2.28(1.32,3.44) | 84.09 | 112 | 105230 | 1433 | | 1.12(0.94,1.30) | 81.79 |  |  |
| HPV 33 | 38 | 7989 | 417 | 5.03(3.69,6.54) | 85.81 | 120 | 104223 | 1648 | | 1.43(1.20,1.68) | 88.54 |  |  |
| HPV 35 | 29 | 5940 | 66 | 0.74(0.32,1.29) | 62.57 | 89 | 94464 | 1025 | | 0.93(0.74,1.15) | 88.28 |  |  |
| HPV 39 | 32 | 6796 | 373 | 5.06(3.72,6.57) | 83.21 | 105 | 101540 | 2175 | | 2.12(1.84,2.42) | 86.68 |  |  |
| HPV 45 | 37 | 7401 | 224 | 2.77(1.88,3.80) | 79.50 | 103 | 98012 | 643 | | 0.52(0.42,0.63) | 67.64 |  |  |
| HPV 51 | 34 | 6967 | 361 | 4.37(3.00,5.97) | 86.88 | 110 | 109964 | 3699 | | 2.78(2.41,3.17) | 92.37 |  |  |
| HPV 52 | 38 | 7936 | 630 | 8.08(6.46,9.85) | 84.76 | 130 | 119354 | 5741 | | 3.95(3.48,4.45) | 93.83 |  |  |
| HPV 56 | 31 | 6336 | 246 | 2.92(1.78,4.28) | 85.59 | 107 | 104614 | 2351 | | 1.66(1.39,1.95) | 91.25 |  |  |
| HPV 58 | 38 | 7865 | 464 | 5.70(4.26,7.31) | 85.94 | 127 | 108651 | 3826 | | 3.26(2.91,3.63) | 88.86 |  |  |
| HPV 59 | 29 | 6022 | 244 | 3.00(1.77,4.48) | 87.07 | 113 | 113949 | 2774 | | 1.93(1.66,2.22) | 90.36 |  |  |
| LR genotypes | |  |  |  |  |  |  | |  | | |  |  |
| HPV 6 | 41 | 8633 | 1678 | 20.57(16.31,25.16) | 95.96 | 126 | 104712 | 19878 | | 18.92(16.46,21.52) | 99.04 |  |  |
| HPV 11 | 40 | 8331 | 1086 | 14.28(10.96,17.94) | 94.84 | 124 | 105012 | 13154 | | 13.43(11.53,15.44) | 98.81 |  |  |
| HPV 42 | 25 | 5549 | 122 | 2.08(1.22,3.13) | 78.48 | 86 | 85470 | 2827 | | 2.23(1.80,2.69) | 93.92 |  |  |
| HPV 43 | 18 | 3562 | 114 | 3.06(1.51,5.05) | 86.18 | 94 | 92263 | 3377 | | 2.69(2.19,3.23) | 95.23 |  |  |
| HPV 53 | 28 | 6027 | 206 | 2.67(1.47,4.17) | 88.47 | 90 | 88532 | 2783 | | 2.34(2.01,2.69) | 89.00 |  |  |
| HPV 61 | 16 | 3111 | 111 | 2.81(1.35,4.69) | 83.70 | 19 | 20405 | 529 | | 2.02(1.45,2.67) | 84.36 |  |  |
| HPV 66 | 35 | 7092 | 239 | 3.13(2.11,4.31) | 82.10 | 97 | 98670 | 2124 | | 1.82(1.55,2.11) | 88.47 |  |  |

***Appendix 8: Prevalence of any HPV among males in different age groups in China, by population group***

| age group | prevalence | study number | positive number | male number (n/%) |
| --- | --- | --- | --- | --- |
| outpatients |  |  |  |  |
| ≤20 | 56.01(49.14-62.79) | 51 | 1423 | 2771(4.37) |
| 21-30 | 50.08(46.18-53.98) | 55 | 10308 | 22252(35.06) |
| 31-40 | 48.1(43.33-52.88) | 65 | 9015 | 21541(33.94) |
| 41-50 | 48.31(42.51-54.12) | 60 | 4314 | 10422(16.42) |
| ≥51 | 52.6(47.01-58.17) | 60 | 3147 | 6484(10.22) |
| health checkups |  |  |  |  |
| ≤20 | - | - | - | - |
| 21-30 | 0.58(0.00-2.62) | 3 | 3 | 222(23.87) |
| 31-40 | 5.01(1.42-10.18) | 4 | 20 | 475(51.08) |
| 41-50 | 0.22(0.00-5.25) | 2 | 4 | 74(7.96) |
| ≥51 | 1.26(0.35-4.47) | 1 | 2 | 159(17.10) |

***Appendix 9: Sensitivity analysis results***

|  | pooled | 95%CI | *I*^2^ | *P* |
| --- | --- | --- | --- | --- |
| Omitting HIV+MSM (outpatients) | 51.94 | 49.01-54.86 | 99.39% | ＜0.001 |
| Total estimate (outpatients) | 52.45 | 49.54-55.35 | 99.39% | ＜0.001 |
